# A novel bi-objective model of cold chain logistics considering location-routing decision and environmental effects

**DOI:** 10.1371/journal.pone.0230867

**Published:** 2020-04-09

**Authors:** Longlong Leng, Jingling Zhang, Chunmiao Zhang, Yanwei Zhao, Wanliang Wang, Gongfa Li

**Affiliations:** 1 Key Laboratory of Special Equipment Manufacturing and Advanced Processing Technology, Ministry of Education, Zhejiang University of Technology, Hangzhou, China; 2 College of Computer Science, Zhejiang University of Technology, Hangzhou, China; 3 Key Laboratory of Metallurgical Equipment and Control Technology, Ministry of Education, Wuhan University of Science and Technology, Wuhan, China; National Taiwan University of Science and Technology, TAIWAN

## Abstract

Economic, environmental, and social effects are the most dominating issues in cold chain logistics. The goal of this paper is to propose a cost-saving, energy-saving, and emission-reducing bi-objective model for the cold chain-based low-carbon location-routing problem. In the proposed model, the first objective (economic and environmental effects) is to minimize the total logistics costs consisting of costs of depots to open, renting vehicles, fuel consumption, and carbon emission, and the second one (social effect) is to reduce the damage of cargos, which could improve the client satisfaction. In the proposed model, a strategy is developed to meet the requirements of clients as to the demands on the types of cargos, that is, general cargos, refrigerated cargos, and frozen cargos. Since the proposed problem is NP-hard, we proposed a simple and efficient framework combining seven well-known multiobjective evolutionary algorithms (MOEAs). Furthermore, in the experiments, we first examined the effectiveness of the proposed framework by assessing the performance of seven MOEAs, and also verified the efficiency of the proposed model. Extensive experiments were carried out to investigate the effects of the proposed strategy and variants on depot capacity, hard time windows, and fleet composition on the performance indicators of Pareto fronts and cold chain logistics networks, such as fuel consumption, carbon emission, travel distance, travel time, and the total waiting time of vehicles.

## 1 Introduction

Logistics, which is a major contributor to carbon emissions (CE), pose challenges to global warming and climate change [1], especially in the context of road transportation. Wang et al. (2017) [2] stated that the CE produced by road transportation accounted for the entire transport sector CE by up to 70% which accounted for 14% in the global CE statistics. In response, according to the report of the United Nation Climate Conference in Copenhagen in 2009, China has promised that the CE would be reduced about 40%–45% of the amount of CE exhausted in 2005 by 2020 [3]. Due to the nature of low-temperature transportation, cold chain logistics are high-consumption and high-emission logistics activities. Hence, the methods should be developed to minimize fuel consumption and CE (FCCE). Moreover, the cold chain is defined as a set of refrigeration steps that maintain the quality and safety of food product [3], which concerns the cost-efficient storage and transportation of temperature-sensitive products [4], which influences the client satisfaction. Hence, given the specific conditions, how to maintain the quality and safety of perishable products is a critical issue for cold chain logistics networks. Therefore, the above two motivated us to define a sustainable network for cold chain considering triple effects: economic, environmental, and social benefits.

In the cold chain logistics networks, there exist several optimization objectives for examining the efficiency of networks, that is, logistics costs, environmental benefit, delivery duration, traveling distance, client satisfaction, the freshness of products, etc. This paper investigated a variant of the cold chain logistics: low-carbon cold chain logistics (LCCC), which is based on a model of the location-routing problem (LRP), hence we call it as LRP-based LCCC (LRPLCCC). In the proposed problem, a bi-objective model was developed by minimizing the total costs consisting of three parts: fixed costs of depots to open, costs of vehicles to rent, and travel costs, where the latter can be defined as the costs of FCCE, and to minimize the total damage of quality. The former is used to reduce the logistics costs (economic effect) and the FCCE (environmental effect), while the latter is modeled to improve client satisfaction (social effect). Moreover, three types of cargos which must be delivered to and picked up from clients are developed: general cargo (GC), refrigerated cargo (RC), and frozen cargo (FC). The GCs can be stored at room or cold temperature (e.g., 3~10, which is used for the RC), while the FC must be preserved at freezing temperature (e.g., -4~-24) in freezing chamber. Hence, the states of delivery vehicles should consist of three functions for meeting the demands of cargos. Aiming at solving the proposed bi-objective model, we proposed a practical and efficient framework which is embedded into seven well-known MOEAs, that is, bi-goal evolution (BiGE) [5], nondominated sorting genetic algorithm- II (NSGA-II) [6], strengthen Pareto evolutionary algorithm2 (SPEA2) [7], nondominated sorting and local search (NSLS) [8], grid-based evolutionary algorithm (GrEA) [9], indicator-based evolutionary algorithm (IBEA) [10], and NSGA-III [11]. The main contributions of this paper are as follows:

1. Problem. In the proposed problem, several practical constraints were also considered: simultaneous pickup and delivery, hard time windows, and heterogeneous fleet, where the first two could which could increase client satisfaction and loyalty. Meanwhile, the types of cargos are classified into three modules: GC, RC, and FC. The former two can posit in one vehicle, but the latter must be separately stored in one vehicle. Moreover, the cold chain logistics were based on LRP.
2. Model. A novel bi-objective model was developed for the proposed problem considering three effects. The first objective can simultaneously concern two effects, which is more fit for practical logistics compared to the logistics using travel distance and time as travel costs. As the above mentioned, the second objective is modeled by the amount of damage in RC and FC (except for GC), aiming at improving the client satisfaction which could be further improved by another level: hard time windows.

To the best of our knowledge, the proposed model for the cold chain has not been studied thus far. The rest of this paper is structured as follows: Section 2 provides a review of related literature; Section 3 defines the formulation for the LRPLCCC considering simultaneous pickup and delivery, heterogeneous fleet, and hard time windows; Section 4 provides the solution method, solution representation, and search operators; Section 5 describes the computational experiments and simulated results; and, Section 6 outlines the study conclusions.

## 2 Literature review

Much attention and efforts have been drawn to the development of effective tools in supply chains and logistics systems [12], such as traveling salesman problem [13], vehicle-routing problem (VRP) [14,15], and LRP [16], where the latter is integrated logistics. Since the LRP was proposed by Jacobsen and Madsen (1980) [17] and Madsen (1983) [18], which deals with the combination of two types of decisions that often arise in logistics: the location of facilities and VRP [16], several LRP variants have appeared in the literature, such as capacitated LRP [16], LRP with simultaneous pickup and delivery [12], LRP with time windows [19], LRP with a heterogeneous fleet [19], etc. The works about LRP are plentiful with many exact and metaheuristic methods for tackling the variants of LRP.

The reason why LRP has been a research hotspot so far is its wide-range practicability and considerable difficulty. This paper investigates the application of LRP in the cold chain considering environmental effects and client satisfaction, hence, the following sections focus on the models of FCCE, low-carbon LRP (LCLRP), and cold chain with its variants based on VRP and LRP.

### 2.1 Models estimating fuel consumption

In the traditional models of logistics, travel distance and time were always used as the routing cost. However, in reality, the routing cost should be the costs of FCCE, which is much more practical. Hence, the models estimating the amount of FCCE should be developed for the logistics transportations. As stated by Demir et al. (2011) [20], measuring and reducing emissions requires good estimations to be fed into planning activities, which in turn require estimation models to be incorporated into the planning activities. Hence, it is necessary to analyze the effects of factors on the amount of FCCE. Demir et al. (2014) [21] provided the classification schemes for the factors: vehicle, environment, traffic, driver, and operations. However, factors of environment, traffic, and driver are quite difficult to obtain and evaluate. Hence, several researchers proposed some models using those factors that can be easily obtained, such as the models proposed by Xiao et al. (2012) [22] and Poonthalir and Nadarajan (2018) [23]. However, the accuracy estimating FCCE of the above models is unsatisfactory.

The above models estimating FCCE could be called as factor models, which are simple FCCE methods by using factors that can be easily obtained, such as load, distance, fuel efficiency, etc. Demir et al. (2014) [21] also summarized much more complicated models estimating FCCE: macro and micro models. The former uses average aggregate network parameters to estimate network-wide emission rates, which means that the factors affecting the FCCE keep constant, while the micro ones estimate the instantaneous vehicle FCCE rates at a more detailed level, which means that some factors could change in seconds like speed. The examples of macro ones are the methodology for calculating transportation emissions and energy consumption [24], the computer programme to calculate emissions from road transportation [25], etc. And the classical models in micro models are the comprehensive modal emission model (CMEM) [26], the models defined by Bowyer et al. (1985) [27], etc.

From the level of complexity, micro/macro views are complicated versions of macro/factor, and the above three are convertible, since some parameters in factor models and macro ones can be viewed as the specific parameters that combine the vehicle-related and average speed, and the dynamic parameters in micro models could be used as average values for simplifying the calculation of FCCE. Hence, the above three models could be classified into two modules: static and dynamic models. Moreover, in terms of estimating mechanism, they could be grouped into the load (power)-based and the regression-models. From the perspectives of estimated accuracy, the micro ones are the best, followed by macro ones, and factor model is the worst, the reason is that the estimation of FCCE depends on plentiful parameters which may change over traveling time. For a comprehensive view of the models and factors, the reader is referred to the surveys [21] and papers [20, 28–31].

### 2.2 LRP considering environmental effects

With the implementation of energy conservation and emission reduction, more and more experts have indicated that logistics should not only consider economic indicators but also can reduce energy and emissions to provide a better ecological environment. Lin et al. (2014) [32] provides a comprehensive survey of VRPs considering FCCE. In their paper, a classification was proposed for various variants of VRP and solution methods. However, the models estimating FCCE were not reviewed, and they only listed and analyzed the papers about VRP considering FCCE. Moreover, as an extensive variant of VRP, the investigation of the green/low-carbon LRP was not provided. Our previous work [29, 30] provided a detailed assessment of the FCCE model for green/low carbon LRP, the solution approach, and the number of optimization objectives. This section provides additional papers on the recently published LCLRP, and other papers are also available in our previous paper [29, 30].

In the last few months, several researchers have provided new sights in LCLRP. Dukkanci et al. (2019) [33] provided a single-objective model defined by logistics costs consisting fixed cost of depots and fuel consumption costs, while no fixed costs of vehicles were optimized. In their model of estimating fuel consumption, the micro view CMEM were used. However, no extra constraints were analyzed like the proposed ones in this paper. Koc (2019) [34] provided a single-objective model consisting of costs of depots, vehicles, and fuel consumptions, like their work published in 2016 [35]. Li et al. (2018) [36] proposed a many-objective model for a variant of LRP, that is, multi-depot green vehicle routing problem. In their paper, the objectives were revenue, logistics cost, traveling time, and carbon emission, where the latter used a simple estimation of carbon emission like the total traveling distance, hence, the reduction in carbon emission was not enough better than those using travel distance and time as traveling cost.

### 2.3 Cold chain logistics

Cold chain logistics is a low-temperature transport that helps improve the preservation of goods such as table grapes [37, 38], salmon delivery [39], vaccine [40] and blood [41]. Therefore, the cold chain stream consumes more fuel and emits more emissions to maintain the quality and safety of perishable goods. Therefore, it is necessary to save energy and reduce carbon emissions in cold chain logistics to seek a win-win situation of economic and environmental sustainability [2].

Recently, the cold chain logistics considering FCCE has received increasing attention from research. Details are as follows:

1. Wang et al. (2017) [2] investigates the optimization of VRP with time windows for the low-carbon cold-chain logistics based on carbon tax in China. In their paper, the objective consists of six parts: fixed costs of vehicles, transportation cost, refrigeration costs, penalty costs (soft time window), damage cost, and carbon emission.
2. Wang et al. (2018) [42] formulated a single-objective LCLRP for the cold chain, and the fixed cost of depots to open was added in the total costs consisting of the above six parts.
3. Qin et al. (2019) [43] modeled a bi-objective model for the cold chain (VRP) considering three effects: total cost, client satisfaction, and carbon emissions.
4. Zhang et al. (2019) [44] proposed a low-carbon VRP-based model for the cold chain, which was very similar to the model proposed by Wang et al. (2017) [2]. The solution method in their paper is an acid-ant colony optimization algorithm.

The above four paper investigated the cold chain considering environmental effects. However, they have several common characteristics: (1) same FCCE model, that is, the factor model proposed by Xiao et al. (2012) [22]; (2) Refs. [2,42,44] applied the travel distance as the routing cost and used penalty costs (i.e., soft time windows), only the Ref. [43] used the FCCE costs as routing cost and did not use the penalty costs; (3) Swam heuristic methods. Refs. [2,42,43] designed the genetic algorithm and Refs. [44] applied an acid-ant colony optimization algorithm; (4) Refs. [2,43,44] defined the model for the cold chain logistics based on VRP, only the Ref. [42] is based on LRP.

However, in the reality, the routing cost is the FCCE cost like the models [34,35], and the factor model estimating FCCE might result in selecting the unpleasant routes with high pollution and high emissions since this model neglect the impacts of speed traveled over each arc and other parameters affecting FCCE. Furthermore, it is known through optimization experience that genetic algorithms and ant colony algorithms are not satisfactory for LRP and VRP. Since VRP and LRP are discrete combinatorial optimization problems, it is difficult to obtain the performance of the solution method by the ant colony algorithm and the discrete steps in the crossover and mutation processes.

The difference between this paper and the above papers are as follows: (1) model. This paper defined a novel bi-objective model for the LRPLCCC with minimizing the total logistics costs and the total amount of damage. The first objective consists of three parts: fixed costs of depots to open, fixed costs of vehicles to rent, and costs of FCCE used as routing cost, and the second objective combined hard time windows are used to improve the quality and safety of food product to improve the client satisfaction; (2) Constraints. The proposed model is limited by three practical constraints, which are first used in the cold chain. (3) Complexity. The above four papers only focus on a single type of cargos, but this paper studies the mixed cargo combined with integrated logistics and cold chain, which is the first study in the mixed logistics. (4) solution method. This paper applies local search and mutation procedures to solve the proposed model and develops an effective framework.

As for the cold chain only considering economic effects were studied by several researchers, and the reader is referred to the Refs. [45–49] based on VRP and Refs. [50, 51] based on LRP, which are the latest papers after 2013, and the earlier works can be seen in the paper of Wang et al. (2018) [42].

## 3 Mathematical model

### 3.1 Problem description

The problem is defined on a complete and directed graph *Ω* = (*V*, *E*), where *V* consists of *N* clients and *M* candidate depots, and the *E* is an edge set. Each client *i*∈*N* have pickup demand *p*_*i*_ and delivery demand *d*_*i*_, and hard time window and service time are (*e*_*i*_, *l*_*i*_) and *st*_*i*_, respectively. Besides, the cargo types are *ξ* = {GC, RC, FC}. Each candidate depot *j*∈*M* has a capacity *CD*_*j*_, a renting fee *FD*_*j*_, and a time windows *DTW*_*j*_. The clients are served by a heterogeneous fleet *H* = {*h*_1_, *h*_2_, *h*_3_}, and each vehicle type have corresponding parameters (see Section 5.2) which are used to estimate the amount of FCCE, including fixed cost *FV*_*h*_ (*h*∈*H*) and a capacity *CV*_*h*_. In terms of edge set, there are a travel distance *D*_*ij*_ and speed limitation *SP*_*ij*_ for each edge (*i*, *j*)∈*E =* {(*i*, *j*): *i*, *j*∈*V*, *i*≠*j*}\{(*i*, *j*): *i*, *j*∈*M*}. The goal is to determine the set of depots to open and the tracing of the routes to minimize the total logistics cost and the amount of damage on the quality of the cargos.

Before defining our model, several assumptions should be described:

1. Each client can only be served once by one vehicle and depot;
2. Each client has the same type of delivery and delivery demands;
3. The GC and RC can be delivered or picked up by the same vehicle for the clients, but the vehicle state must adaptively change the state of the refrigerator according to the type of cargo on each edge, and the FC must be supplied in the vehicle in the frozen state;
4. Each vehicle must return to the original depot before the depot closes;
5. The load of each vehicle on each edge must be less than its capacity;
6. The depot must serve the clients assigned to it;
7. The load of each depot must be less than its capacity;
8. The vehicles travel on each edge with the known speed limitation without considering the road and traffic conditions;
9. The vehicle must wait until the moment reaches the opening time window of each client if it arrives early.

### 3.2 Model development

This section provides the model estimating FCCE and a variable function of refrigerated goods quality.

1. Model estimating FCCE

This section presents a microscopic model to estimate the amount of FCCE, namely CMEM, which is easily applicable to calculate FCCE, and it has been extensively used in LCLRP [28–30, 34, 35]. This paper applies the version of CMEM which is used in the cases with a heterogeneous fleet. The fuel consumption rate *FCR*_*h*_ (g/s) of a vehicle type *h*∈*H* is given by

$$FCR_{h}=\varphi \left(k_{h}N_{h}T_{h}+P_{h}/\eta \right)/\kappa$$ (1)

where *φ* is the fuel-to-air mass ratio; *k*_*h*_ is engine fiction parameter (kJ/rev/L); *N*_*h*_ is engine speed (rev/s); *T*_*h*_ is engine displacement (L); *P*_*h*_ is the second-by-second engine power putout (in kW); *η* is an efficiency parameter for diesel engines; and *κ* is the heating value of a typical diesel fuel (kJ/g).

$$P_{h}=P_{h}^{rc}+P_{h}^{tract}/n_{tf}$$ (2)

where *P*_*h*_^*tract*^ represents the total tractive power putout (kW); *η*_*tf*_ is the vehicle drive train efficiency; *P*_*h*_^*rc*^ is the engine power demand associated with running losses of the engine and the operation of vehicle accessories such as air conditions and refrigerating compressor in the cold chain logistics. The estimation of *P*_*h*_^*tract*^ is given by:

$$P_{h}^{tract}=\left(Ga+Gg\sin\theta +0.5C_{h}^{d}\rho A_{h}s{\left(t\right)}^{2}+GgC_{r}\cos\theta \right)\times s(t)/1000$$ (3)

where *G* is the sum of the total weight of the vehicle weight (*w*_*h*_) and the vehicle load (kg); *a* and *g* are acceleration of vehicle and gravitation (m/s^2^), respectively; *θ* is the road angle; *C*_*h*_^*d*^ and *C*_*r*_ are, respectively, coefficient of aerodynamic drag of a type *h*∈*H* and coefficient of rolling resistance; *ρ*/*A*_*h*_ is the air density (kg/m^3^)/the front surface area (m^2^); *s* is the instantaneous traveling speed (m/s). Then the fuel consumption *F*_*h*,*g*_ (g) of vehicle type *h* over traveling a time *t* from *t*_1_ to *t*_2_ at instantaneous traveling speed *s*(*t*) is calculated as

$$F_{h,g}=\int_{t_{1}}^{t_{2}}FCR_{f}\mathrm{dt}=\varphi k_{h}N_{h}T_{h}\left(t_{2}-t_{1}\right)/\kappa +\frac{\varphi }{\eta \kappa }\left(P_{h}^{rc}\left(t_{2}-t_{1}\right)+\left(\int_{t_{1}}^{t_{2}}P_{h}^{tract}\mathrm{dt}\right)/n_{tf}\right)$$ (4)

$$\int_{t_{1}}^{t_{2}}P_{h}^{tract}\mathrm{dt}=\left(G\left(a+g\sin\theta +gC_{r}\cos\theta \right)\int_{t_{1}}^{t_{2}}s\left(t\right)\mathrm{dt}+0.5C_{h}^{d}\rho A_{h}\int_{t_{1}}^{t_{2}}s{\left(t\right)}^{3}\mathrm{dt}\right)/1000$$ (5)

$$F_{h,L}=F_{h,g}/\psi =\lambda \left(\left(k_{h}N_{h}T_{h}+P_{h}^{rc}/\eta \right)\times \left(t_{2}-t_{1}\right)+\gamma wG\int_{t_{1}}^{t_{2}}s\left(t\right)\mathrm{dt}+\gamma \beta_{f}\int_{t_{1}}^{t_{2}}s{\left(t\right)}^{3}\mathrm{dt}\right)$$ (6)

where *λ = φ/κψ*, *γ =* 1*/(*1000*×n*_*tf*_*η)*, *w = a+g*sin*θ+gC*_*r*_cos*θ* and *β*_*v*_ *=* 0.5*C*_*h*_^*d*^*ρA*_*h*_, and *ψ* is conversion factor which converts *F*_*h*,g_ in gram to *F*_*h*,L_ in liter. The above microscopic equation is a basic estimate of FCCE and consists of three modules: (1) engine module, linearly proportional to the travel time; (2) moment module, linearly proportional to the product of vehicle weight and travel distance; (3) speed module, linearly proportional to the integral of *s*(*t*)^3^. If a vehicle of type *h* travels a distance *d* at a constant speed *s*, then Eq (6) can be rewritten as:

$$F_{h,L}=\lambda \left(\left(k_{h}N_{h}T_{h}+P_{h}^{rc}/\eta \right)\times d/s+\gamma wG\times d+\gamma \beta_{h}ds^{2}\right)$$ (7)

Eq (7) is the general model estimating the fuel consumption during *s*≠0. However, for RC and FC, the vehicles must wait for the clients until the opening time window is open. Hence, the refrigerating compressor must run for keeping the quality and safety of food production during the waiting time of vehicles and service time for RC and FC. Therefore, for the cases in this paper, the *F*_*ijh*_ over arc (*i*, *j*)∈*E* can be written as

$$\begin{array}{c} F_{ijh}=\lambda \left(k_{h}N_{h}T_{h}+P_{h}^{rc}\left(dv1_{ij}\right)/\eta \right)\times \left(D_{ij}/SP_{ij}+\left(\max\left\{e_{i}-AT_{ih}\right\}+st_{i}\right)\times dv2_{ij}\right)x_{ijh}\\ +\lambda \gamma w\left(L_{ijh}+w_{h}\right)D_{ij}x_{ijh}+\lambda \gamma \beta_{h}D_{ij}{\left(SP_{ij}\right)}^{2}x_{ijh} \end{array}$$ (8)

where

*dv*1_*ij*_ is the state variant of the refrigerating compressor depending on type of cargos (see Eq 9), indicating that each type of vehicle has three states.

*AT*_*ih*_ is the arriving moment on the node *i*∈*V* of a vehicle type *h*;

*L*_*ijh*_ is the load of vehicle type *h* over arc (*i*, *j*)∈*E*;

*dv*2_*ij*_ is a variant equaling to 1 if the RC/FC is a part of *L*_*ijh*_ and to 0 otherwise.

*x*_*ijh*_ is a decision variant which equals to 1 if a vehicle of type *h*∈*H* travels on edge (*i*, *j*)∈*E* and to 0 otherwise.

$$dv1_{ij}=\left\{ \begin{array}{l} 1,\;\mathrm{if}\;L_{ijh}\;\mathrm{is}\;\mathrm{GC}\\ 2,\;\mathrm{if}\;L_{ijh}\;\mathrm{is}\;\mathrm{mixed}\;(\mathrm{GC}\;\mathrm{and}\;\mathrm{RC})\;\mathrm{or}\;\mathrm{RC},\forall i,j\in V\\ 3,\;\mathrm{if}\;L_{ijh}\;\mathrm{is}\;\mathrm{FC} \end{array}\right.$$ (9)

The assumption H3 is imposed by *dv*1_*ij*_×*dv*1_*jk*_∈{1,2,4,9} if the clients *i*, *j*, and *k* are served by the same vehicle. Moreover, as reported by Refs. [28–30,34,35], the exhausted CE (in kilogram) is estimated through the amount of fuel consumption, in other words, the amount of carbon emission is directly proportional to the amount of fuel consumption, namely, 1 liter of fuel can produce 2.32 kilogram of CO_2_. Hence, the cost of FCCE is as follows:

$$C_{ijh}=\left(c_{fc}+c_{cc}\times 2.32\right)\times F_{ijh}$$ (10)

where *c*_*fc*_ and *c*_*cc*_ are, respectively, the price of 1-L fuel and 1-kg CE.

1. Damage on quality

This paper applies the variable function of the quality of refrigerated goods used in [2, 42–44]: *D*(*t*) = *D*_0_*e*^–*at*^, where *D*(*t*) is the quality of the cargo at time *t*; *t* is the transportation time; *D*_0_ is the quality of the product from the depot; and *a* is, related to the characteristics of cargos and temperature, the spoilage rate of the product. Combined with the characteristics of this paper, we used the following equations to calculate the amount of damage on the quality:

$$D1_{ijh}=L_{ijh}^{*}\times \left(1-e^{-a_{1,lx}\left(AT_{jh}-\max\left\{e_{i},AT_{ih}\right\}-st_{i}+\left(\max\left\{e_{j}-AT_{jh},0\right\}+st_{j}\right)dv2_{ij}\right)}\right)$$ (11)

$$D2_{ijh}=\left(L_{ijh}^{*}-q_{j}\times r\times x_{ijh}\right)\times \left(1-e^{-a_{2,lx}st_{j}dv2_{ij}}\right)$$ (12)

where

*D*1_*ijh*_ is the damage when the vehicle *h*∈*H* leaves node *i*∈*V* and arrives at node *j*∈*V* and the door is not opened in the process of transportation;

*D*2_*ijh*_ is the damage when the vehicle *h*∈*H* serves client *j*∈*V* (the door is open);

$L_{ijh}^{*}$ is the RC/FC weight of the vehicle *h*∈*H* over arc (*i*, *j*)∈*E*, if the total load belongs to GC, its value equals to 0;

*a*_1,*lx*_ and *a*_2,*lx*_ are the spoilage rates for the cargos type *lx*∈{GC, RC, FC}, respectively;

Let *r* equal to 0 if the cargos type of client *j*∈*V* is GC and to 1 otherwise.

Hence, the total damage on the quality of cargos are as

$$D_{ijh}=D1_{ijh}+D2_{ijh}$$ (13)

### 3.3 Establishment of the formulation

Section 3.2 provides a model for estimating FCCE and a method for calculating quality damage. Therefore, the formal model of LRPLCCC can be defined as follows:

$$\min\;TotalCost=\sum_{i\in M}FD_{i}y_{i}+\sum_{i\in M}\sum_{j\in N}\sum_{h\in H}FVx_{ijh}+\sum_{i\in V}\sum_{j\in V}\sum_{h\in H}C_{ijh}x_{ijh}$$ (14)

$$\min\;TotalDamage=\sum_{i\in V}\sum_{j\in V}\sum_{h\in H}D_{ijh}$$ (15)

where *y*_*i*_ be equal to 1 if a depot *i*∈*M* is selected and to 0 otherwise. Objective (14) is the total cost of three parts: fixed costs of the open depots, the fixed costs of the leased vehicles, and the FCCE costs. Objective (15) is the total damage of RC and FC quality.

The following constraints are primarily used to satisfy and guarantee the above hypotheses in Section 3.1. The following two are degree restrictions. In particular, constraint (16) states that each customer must serve only once; constraint (17) provides a balance between entering arcs and leaving arcs.

$$\sum_{i\in V}\sum_{h\in H}x_{ijh}=1,\forall j\in N$$ (16)

$$\sum_{i\in V}\sum_{h\in H}x_{ijh}=\sum_{i\in V}\sum_{h\in H}x_{jih},\forall j\in N$$ (17)

The following three ensure that a load on the depot must not exceed its capacity and the additional restrictions on the depot load at the beginning and end of the service.

$$\max\left\{\sum_{i\in N}d_{i}z_{ij},\sum_{i\in N}p_{i}z_{ij}\right\}\leq CD_{j}y_{j},\forall j\in M$$ (18)

$$\sum_{i\in N}\sum_{h\in H}L_{jih}=\sum_{i\in N}d_{i}z_{ij},\forall j\in M$$ (19)

$$\sum_{j\in N}\sum_{h\in H}L_{jih}=\sum_{j\in N}p_{j}z_{ji},\forall i\in M$$ (20)

where *z*_*ij*_ indicates the assignment of the client *i*∈*N*, if it is assigned to the depot *j*∈*M*, then *z*_*ij*_ = 1, otherwise *z*_*ij*_ = 0. Corresponding constraints on a load of vehicles on each edge are as follows:

$$0\leq L_{ijh}\leq CV_{h}x_{ijh},\forall i\in V,j\in V,h\in H$$ (21)

$$\left(d_{i}-p_{i}\right)x_{jih}\leq L_{jih}\leq \left(CV_{h}-d_{i}+p_{i}\right)x_{jih},\forall i\in N,j\in V,h\in H$$ (22)

$$\sum_{i\in M}\sum_{j\in N}L_{ijh}=\sum_{i\in N}\sum_{j\in V}d_{i}x_{ijh},\forall h\in H$$ (23)

$$\sum_{i\in N}\sum_{j\in M}L_{ijh}=\sum_{i\in N}\sum_{j\in V}p_{i}x_{ijh},\forall h\in H$$ (24)

$$\sum_{i\in V}\sum_{h\in H}\left(L_{ijh}-d_{j}\right)x_{ijh}=\sum_{i\in V}\sum_{h\in H}\left(L_{jih}+p_{j}\right)x_{jiv},\forall j\in N$$ (25)

In detail, constraint (21) imposes that the load on each edge must be less than the capacity of the assigned vehicle; constraint (22) is the bound on the load variables; constraint (23) and (24) are the restrictions on the load variables at the starting and finishing stage; constraint (25) implies that the pickup and delivery demand of each client is met.

The constraints on hard time window for each client and each depot are as follows:

$$AT_{jh}=\left(\max\{AT_{ih},e_{i}\}+st_{i}+D_{ij}/SP_{ij}\right)x_{ijh},i\in V,j\in V,h\in H$$ (26)

$$AT_{ih}x_{ijh}\leq AT_{jh}x_{jkh}\leq l_{j},\forall i\in V,j\in N,k\neq i,h\in H$$ (27)

$$AT_{jh}\leq DTW_{j},\forall j\in M,h\in H$$ (28)

Eq (26) is the continuity of the vehicle travel time. Constraint (27) stipulates that the arrival time of each vehicle must not exceed the closing time window of each client. Constraint (28) limits each vehicle to return before the closing time of each depot. The following three prohibits the formation of routes that do not start and end in the same depot:

$$\sum_{h\in H}x_{ijh}\leq z_{ij},\forall i\in N,j\in M$$ (29)

$$\sum_{h\in H}x_{ijh}\leq z_{ji},\forall j\in N,i\in M$$ (30)

$$\sum_{h\in H}x_{ijh}+z_{ik}+\sum_{m\in M,m\neq k}z_{jm}\leq 2,\forall i,j\in N,k\in M$$ (31)

Constraints (32) and (33) guarantee that each client must be served by only one vehicle and depot to open:

$$\sum_{j\in M}z_{ij}=1,\forall i\in N$$ (32)

$$x_{ijh}+\sum_{k\in V}\sum_{p\in H,p\neq h}x_{jkp}\leq 1,\forall i\in V,j\in N,h\in H$$ (33)

Constraints (34) and (35) make sure that the depot to open must serve at least one client:

$$z_{ij}\leq y_{j},\forall i\in N,j\in M$$ (34)

$$\sum_{i\in N}z_{ij}\geq y_{j},\forall j\in M$$ (35)

In particular, constraint (34) indicates that the unselected depots must not serve any of the clients; constraint (35) states that the depot to be opened must serve at least one client.

### 3.4 Extra valid restriction

Constraints (16)–(35) are the key and necessary for the proposed problem, and this paper also provides several polynomial-size, valid, not necessary, and alternative inequalities described below. First, the subtour must not exist, that is, subtour elimination, which could be seen as a complementary one for constraints (16) and (17):

$$x_{ijh}+x_{jih}\leq 1,\forall i,j\in N,h\in H$$ (36)

Constraint (36) is special case of classical subtour elimination constraints used by Koc et al. (2016) [19]. The following inequalities are used to restrict the depots to open and vehicles:

$$\sum_{j\in N}x_{ijh}\leq y_{i},\forall i\in M,h\in H$$ (37)

$$\sum_{i\in N}\sum_{h\in H}x_{ijh}\geq y_{j},\forall j\in M$$ (38)

In particular, constraint (37) indicates that the unselected depot must not assign a vehicle; constraint (38) states that the depots to be opened must allocate vehicles to serve the clients. The above can be used as a supplementary restriction for constraints (16), (17), (34) and (35), since constraints (16), (17), (34) and (35) mandate that each client must serve only once by a vehicle and opened depot, indicating that the open depot must allocate the vehicle.

Constraint (39) is the bound on the number of the assigned vehicles, including upper bound and lower bound.

$$\left\lceil \max\left\{\sum_{i\in N}d_{i},\sum_{i\in N}p_{i}\right\}/\max\left\{CV\right\}\right\rceil \leq \sum_{i\in M}\sum_{j\in N}\sum_{h\in H}x_{ijh}\leq \left|N\right|$$ (39)

where ⌈•⌉ is the smallest integer larger than •. The next inequality imposes the number of depots to open:

$$\sum_{i\in M}CD_{i}y_{i}\geq \max\left\{\sum_{i\in N}d_{i},\sum_{i\in N}p_{i}\right\}$$ (40)

However, constraint (40) is not always feasible, since it also depends on the number of clients, the example is the instance with 55 clients and 15 depots introduced by Barreto et al. (2007) [52]. The final inequality is a complementary restriction for constraint (33) to forbid the different depots in a single route.

$$\sum_{j\in N}x_{ijh}+\sum_{j\in N}x_{jgh}\leq 1,\forall j,g\in M,j\neq g,h\in H$$ (41)

The above constraints (16)–(41) could also be used in our previous works [29–30] for the another variant of LCLRP except for the constraint (28).

## 4 Proposed methods

Since this paper tackles a bi-objective model for the LRPLCCC, the corresponding MOEAs were used to obtain the Pareto solutions. In seven MOEAs, a practical framework was developed to generate the children solutions which is fit to the local search and mutational procedures (Section 4.1). In the proposed framework, a simple and effective chromosome representation for the proposed LRPLCCC were developed (Section 4.2). Moreover, we designed 14 neighborhood operators (Section 4.3). The details are provided as follows.

### 4.1 Framework used in MOEAs for the LRPLCCC

**Fig 1:**
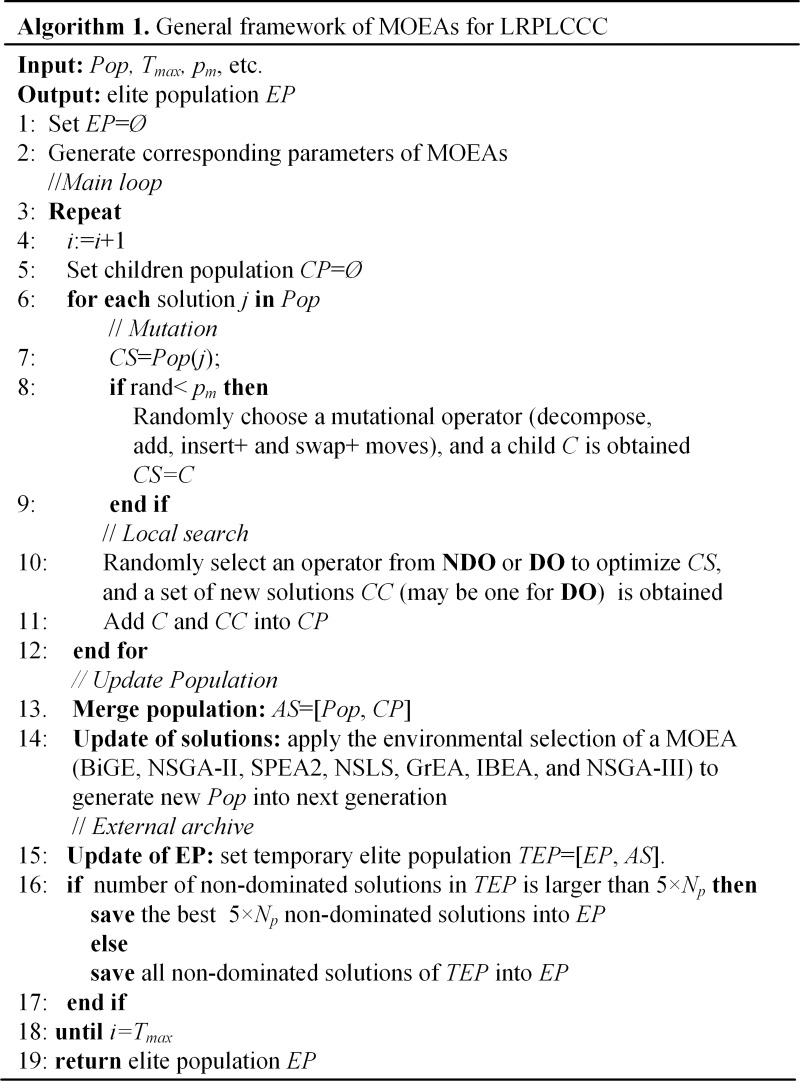
Algorithm 1. General framework of MOEAs for LRPLCCC.

An overview of the pseudocode for the proposed framework used in MOEAs is given in Algorithm 1 (Fig 1). Input data includes the maximum number of iterations (*T*_*max*_), mutation probability (*p*_*m*_), and initial population (*Pop*) made of feasible random chromosomes (Section 4.2). The output data is an elite population (EP) that consists of non-dominated solutions by removing duplicates and dominating solutions.

Step 2 is the parameter settings in the seven MOEAs, such as the grid density in GrEA, the scaling parameter in the IBEA, and the reference points (or uniform points) in the NSGA-III. Then the main loop is performed, stopping when a maximum number *T*_*max*_ of iterations.

In each iteration of the main loop, the evolutionary phase is first performed in Steps 6–12. In other words, for each individual in the current population, a mutational operator is randomly selected from the pool of perturbation to provide slight randomness, depending on the *p*_*m*_ value, then a local search procedure is also chosen from the pool of local search procedures to improve the quality of the obtained solution.

After improving all individuals in the current population, the children population (CP) is merged with the parent population (i.e., the current population) to obtain the next population with *N*_*p*_ individuals by utilizing the update mechanism used in seven MOEAs (Steps 13–14). Moreover, this framework also provides the external archive to save the best non-dominated solutions with 5×*N*_*p*_ individuals which is the data outputted (Steps 16–17).

When the main loop stops, the algorithm ends and returns to EP.

### 4.2 Chromosome representation

Since LRPLCCC is one of the discrete combinatorial optimization problems, a simple and efficient representation is used to represent the solution, which was also used in our previous paper [12, 28–30]. In the construction of solutions, each route (corresponding to the vehicle route) consists of a sequence of clients and depots inserted at both ends. Hence, a complete solution is represented by *R* = {*r*_1_, *r*_2_, …, *r*_*k*_}, where *r*_*i*_ is a complete route. Besides, we also save several attributes, such as the load, type, and state of vehicles and two objective values, aiming at allowing a fast calculation in the implementation process in the operators and objective space. Besides, the adopted representation, together with the operators (detailed in Section 4.2), could provide feasible children solutions by meting constraints (16)–(41).

**Fig 2:**
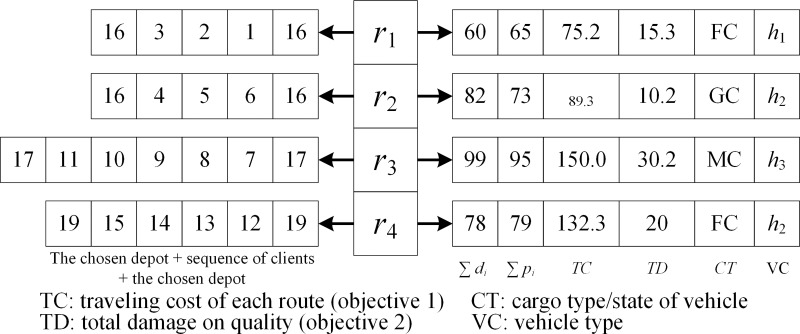
A simple solution representation.

Fig 2 is a simple example of a chromosome representation of LRPLCCC. Four cars were assigned to serve 15 clients, as shown in the left portion, and the attributes of each route are listed in the right portion. Besides, the initial population is randomly generated, which satisfies all constraints (16)–(41).

### 4.3 Neighborhood search mechanisms

In the proposed framework, 14 operators are designed to obtain the Pareto solutions, which can be classified into three modules: dominated operators (DO), Non-dominated operators (NDO), and mutational operators (MO). The execution mechanism of DOs is to obtain the children solutions dominated the parent individuals, which can accelerate convergence in the early stages of the algorithm. Since EP consists of non-dominated solutions, NDOs are used to obtain a large number of non-dominated solutions. While often insufficient to achieve competitive results, MOs can be used to provide randomization when searching globally for performing simple random moves. In this paper, the DO and NDO pools consist of five operators: 2-opt, swap, insert, segment-based swap, and segment-based insert. The MO pool consists of four operators: add, decompose, insert +, and swap+ moves. Details are as follows.

The 2-opt move is executed by removing two edges from two routes and reconnecting the new four paths created (see Refs. 52). The swap move swaps the location of two clients from different routes. The insert move is implemented by moving a client into a position of another route. The segment-based swap and segment-based insert moves like swap and insert moves, but the objects of them are two or three clients instead of one client.

**Fig 3:**
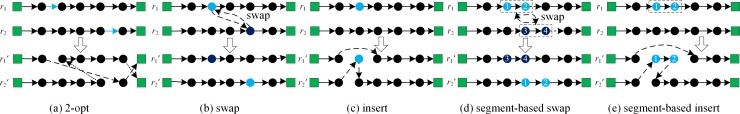
Schematic diagrams of 2-opt, swap, and insert moves.

The schematic diagrams of the above three moves are provided in Fig 3. As to the schematic diagrams of insert+ and swap+ moves also like swap and insert moves, but the output of these moves usually are dominated by the parent solutions.

The above operators are implemented by a complete process [53] rather than random selection, avoiding costing CPU time. Moreover, considering the hard time windows, the schematic diagrams of the above 12 operators implement the moves between different routes rather than those inside the routes.

As to the add move, it seeks to avoid a fast convergence to solutions with few depots (prone to happen due to 2-opt, insert, and segment-based insert moves). Meanwhile, it diversifies the opened depots by opening a new one and randomly reassigning between 1 and 2/3 of the routes to it [16] or randomly choosing a depot from the set of all depots to open after closing one opened depot.

The decompose move is executed by decomposing one route into two routes, and the breakpoint is randomly selected only if it has more than one clients to serve. The benefit is to avoid a fast convergence since the 2-opt, insert, and segment-based insert moves could easily result in the long tracing of the route using vehicles with higher capacity.

Since a forthcoming article (2020) has tested and analyzed the performance of the first found and the best improvement, the results showed that the performance of the operators using the first found improvement is superior to those using the best improvement. Hence, this paper used first found improvements as the mechanism in operators. In other words, if a better solution is found, the operator will stop and return to the child solution. In the DO pool, the definition of "better solution" is that the child solution dominates the parent solution, but in the NDO pool, it is defined that the parent solution cannot be dominated by the child.

## 5 Optimization simulation and analysis of results

Implementation aspects and evaluations of the proposed problem and algorithms are provided and discussed in the following sections.

### 5.1 Implementation aspects and parameters configurations

The proposed algorithms and problem were coded in MATLAB and results were obtained using 4.0 GHz Intel Core i7-6700K CPU with 12 GB of RAM and running Windows 10.

Parameters configuration plays an important role in affecting the performance of the proposed problem and algorithms. However, this paper favors the default values which has tuned by the published papers, such as the scaling factor in GrEA (0.05) [9] and the number of the divisions of the objective space in each dimension (45) [10].

The size of the initial population *N*_*p*_ is 100, just like the traditional setting of the bi-objective problem. And the maximum number of iterations, *T*_*max*_, is directly dependent on the number of nodes (i.e., clients and depots):

$$T_{\max}=\alpha \left(\left|N\right|+\left|M\right|\right)$$ (42)

For all instances, the multiplier *α* is set to 10. As for the size of the external archive, we return 5×*N*_*p*_ elite individuals to prevent the loss of non-dominated solutions during the search process. For the mutational probability *p*_*m*_, we conducted an initial experiment with different values in the interval [0, 1] combined with seven MOEAs. The reason can be drawn that each MOEA may favor different *p*_*m*_. And the results for the initial experiment were presented in Section 5.4.

### 5.2 Test instances

Since the LRPLCCC is first studied in this paper, the instances proposed by the existing papers are unable to be reused. Therefore, we randomly generated 28 instances with different number of clients and depots, called as C|*N|*-|*M|*-*No*., where |*N|* and |*M|* are, respectively, the number of clients and depots, and *No*. is the serial number of instances. The number of clients is |*N|*∈{20,30,40,50,60} and the number of candidate depots is |*M*|∈{4,5,6,7,8}. All nodes are randomly located in the square interval [0, 50]^2^ km. The delivery and pickup demand of each client is generated using a uniform distribution in the range [100, 1600] kg, and the opening time window *e*_*i*_ (*i*∈*N*) of each client is obtained from the instance C101 [54], which decreased by 50%, and the closing time window *l*_*i*_ (*i*∈*N*) for each client depends on the service time (i.e., *l*_*i*_ = *e*_*i*_+*st*_*i*_):

$$st_{i}=1800\times \frac{d_{i}+p_{i}}{\sum_{i\in N}\left(d_{i}+p_{i}\right)/\left|N\right|}$$ (43)

The capacity and fixed cost of each candidate depot are, respectively, generated using a uniform distribution in the range [10, 15] tons and [500, 1000] yuan. And the closing time window for all depots is set to 12 hours.

Regarding the parameters of the heterogonous fleet, they are given in Tables 1 and 2, which provide vehicle-specific parameters and general parameters, including fixed costs, freezer power for each type of vehicle, rate of corruption, etc.

**Table 1:** Parameters in the proposed model.

Notation	Description	Typical values
*φ*	Fuel-to-air mass ratio	1
*η*	Efficiency parameter for diesel engines	0.45
*κ*	Heating value of a typical diesel fuel (kJ/g)	44
*n*_*tf*_	Vehicle drive train efficiency	0.45
*a*	Acceleration (m/s^2^)	0
*g*	Gravitational constant (m/s^2^)	9.81
*θ*	Road angle	0
*ρ*	Air density (kg/m^3^)	1.2041
*C*_*r*_	Coefficient of rolling resistance	0.01
*ψ*	Conversion factor (g/s to L/s)	737
*C*_*cc*_	CO_2_ emissions cost (Yuan/kg)	0.05
*C*_*fc*_	Fuel consumption cost (Yuan/L)	7.5
*ϐ*	Conversion factor (L fuel to kg CO_2_ emission)	2.32
*a*_1_	Spoilage rate when door is closed (GC/RC/FC)	0/0.001/0.002
*a*_2_	Spoilage rate when door is opened(GC/RC FC)	0/0.002/0.003

**Table 2:** Vehicle-specific parameters.

Notation	Description	*h*_1_	*h*_2_	*h*_3_
*w*_*h*_	Curb weight (kg)	3500	4500	5500
*CV*_*h*_	Maximum payload (kg)	4000	7500	12500
*k*_*h*_	Engine friction factor (kJ/rev/L)	0.25	0.23	0.20
*N*_*h*_	Engine speed (rev/s)	38.34	37.45	36.67
*T*_*h*_	Engine displacement (L)	4.5	4.5	6.9
*C*_*h*_^*d*^	Coefficient of aerodynamics drag	0.6	0.64	0.7
*A*_*h*_	Frontal surface area (m^2^)	7.0	7.4	8.0
*FV*_*h*_	Vehicle fixed cost (Yuan per time)	100	70	50
*P*_*h*_^*rc*^	Power of refrigerating GC/RC/FC (kW/s)	0/5/8	0/8/10	0/10/15

### 5.3 Performance metrics

To validate the reliability of the proposed algorithms, the four well-known performance metrics, namely generational distance (GD), inverted generational distance (IGD), hypervolume (HV), and the ratio of non-dominated solutions (RNI), are applied.

GD describes the quality of an approximate Pareto front (APF) by measuring the distance between APF and real Pareto front (RPF), the smaller the GD value, the better the quality. The GD equals to 0 indicates that APF is a part of RPF.

The IGD describes the quality and uniformity of APF by measuring the distance between RPF and APF. The smaller the IGD value, the better the distribution and convergence. The IGD equals to 0 indicates that the APF equals to RPF.

The HV measures the size of the coverage space between the APF and the reference point. The larger the HV value, the better the diversity and distribution. HV is in the range [0, ∞].

RNI describes the contribution rate of the APF for the RPF. The larger the RNI value is, the better the APF. The RNI equals to 1 indicates that the APF equals to RPF.

However, in our case RPF is unknown. For this reason, and following the paper [55], this set RPF was made of all sets APF obtained by all MOEAs by removing dominated and repeated individuals. The set RPF is in fact an approximation of the real Pareto front.

### 5.4 Parameter turning (*p*_*m*_) and analyses on the pairs of MOEAs

The mutational probability *p*_*m*_ has significant impacts on the performance of MOEAs. For the proposed seven MOEAs (BiGE, GrEA, IBEA, NSGAIII, NSGAII, NSLS, and SPEA2), a situation may exist: a special pair of each algorithm and *p*_*m*_ value could maximize performance in obtaining the Pareto solutions. Hence, we evaluated the effects of the *p*_*m*_ values on the performance of seven MOEAs. The instances used in this section were the C20-4-no., C30-5-no., and C40-6-no., where no.∈ {1,2,3,4}, i.e., the total 12 instances. Each MOEA performed four runs on each instance. Moreover, we tested the impacts of the *p*_*m*_ values in the range [0,1], i.e., *p*_*m*_∈ {0, 0.1, 0.2, 0.3, 0.4, 0.5, 0.6, 0.7, 0.8, 0.9, 1}. Therefore, a total of 3696 runs were performed. To rank MOEAs, we ranked 77 pairs using the scoring system of the CHESC-cross-domain heuristic search challenge (http://www.asap.cs.nott.ac.uk/external/chesc2011/). In this system, the top eight pairs score 10, 8, 6, 5, 4, 3, 2 and 1 respectively. The media used are performance metrics (HV, IGD, and GD) obtained from the average of four runs. Therefore, the highest score for each pair is 120 (12 instances). The results are shown in Table 3.

**Table 3:** Scores of 77 pairs evaluated by HV, IGD, and GD values.

MOEA	Medium	0	0.1	0.2	0.3	0.4	0.5	0.6	0.7	0.8	0.9	1
BiGE	HV	0	0	0	0	0	9	20	17	13	0	0
GrEA		0	0	0	3	0	5	2	17	15	1	0
IBEA		0	0	0	4	20	20	8	14	14	4	0
NSGA-III		0	0	4	4	8	11	13	9	2	**25**	0
NSGA-II		0	0	3	3	5	8	17	**49**	33	15	6
NSLS		0	0	0	0	0	6	11	1	0	1	0
SPEA2		0	0	0	0	0	5	13	6	22	2	0
BiGE	IGD	0	0	0	0	0	0	11	14	7	10	4
GrEA		0	0	0	0	0	0	1	0	12	8	0
IBEA		0	0	0	0	0	0	0	6	10	0	5
NSGA-III		0	0	0	0	9	10	8	16	21	9	3
NSGA-II		0	0	10	5	16	6	**25**	19	7	24	18
NSLS		0	3	0	3	0	0	7	14	7	11	6
SPEA2		0	4	6	3	18	3	13	16	17	**33**	10
BiGE	GD	23	**42**	5	0	5	0	5	0	0	0	0
GrEA		20	**47**	23	9	3	9	0	0	1	0	0
IBEA		17	24	8	14	17	7	0	0	0	0	0
NSGA-III		5	18	4	13	10	0	8	0	0	0	0
NSGA-II		7	12	0	0	0	14	0	0	0	0	0
NSLS		25	4	2	0	0	0	3	0	0	0	0
SPEA2		6	12	8	10	19	6	0	0	3	0	0

**Fig 4:**
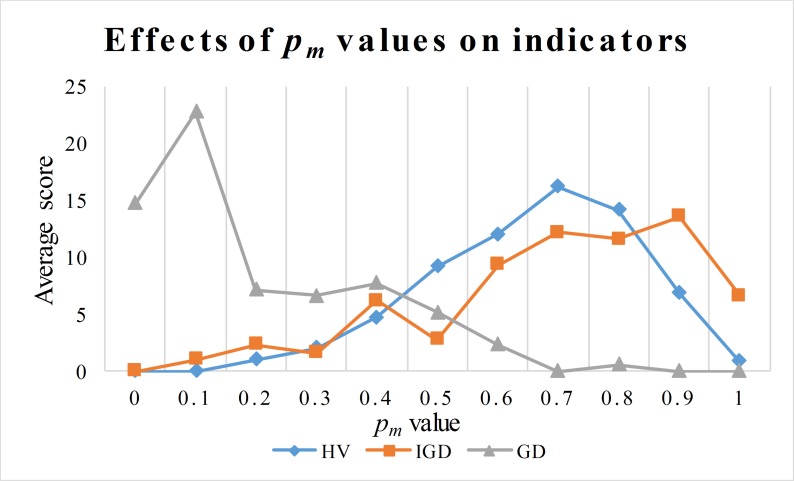
Effects of *p*_*m*_ values on the performance of algorithm.

Looking at Table 3, the first two pairs with the best HV, IGD, and GD are NSGA-II (0.7), NSGA-III (0.9), NSGA-II (0.6), SPEA2 (0.9), BiGE (0.1), and GrEA (0.1). They were used in the following experiments to analyze the effects of problem parameters on performance parameters of the Pareto frontier and the cold chain network. We could find that: (1) The *p*_*m*_ value has a great impact on the performance of MOEAs. In the context of HV values, the proposed MOEAs favor different *p*_*m*_ values. i.e., {0.6, 0.7,0.4/0.5, 0.9,0.7,0.6, 0.8} which are in the range [0.4 0.9]. (2) In terms of average scores (neglecting types of MOEAs), the *p*_*m*_ values are 0.7, 0.9, and 0.1 which can help obtain the best values of HV, IGD, and GD, as shown in Fig 4. (3) Different instances favor different *p*_*m*_ values, but the best *p*_*m*_ values located in the range [0.3,0.9] in terms of the whole performance (HV and IGD), which illustrated in the figures in the S1 File. However, the performance of MOEAs using 0.1 as the *p*_*m*_ value can achieve the best performance in terms of GD values. The reason is that the fewer mutation operators are used, the higher the intensification of the algorithm, but the performance of MOEAs using 0 as the *p*_*m*_ value will seriously deteriorate the performance of the algorithm. Therefore, it is very important to use the perturbation operator in the search process.

Aiming at analyzing the effects of problem parameters on the performance indicators of Pareto fronts and cold chain logistics, we used the top two MOEAs in terms of average HV, IGD, and GD values, that is, NSGA-II (0.7), NSGA-III (0.9), NSGA-II (0.6), SPEA2 (0.9), BiGE (0.1), and GrEA (0.1).

### 5.5 The effect of the cargo type

In this paper, the proposed strategy (S1) is assumed: the load of several vehicles may be mixed cargos (GC and RC), and the FC must be served separately, i.e., *dv*1_*ij*_×*dv*1_*jk*_∈ {1,2,4,9}. At the same time, there is another version (S2): the type of cargos loaded in each vehicle must keep the same, in other words, each vehicle only loads one type of cargos, i.e., *dv*1_*ij*_×*dv*1_*jk*_∈ {1,4,9}. To analyze these two strategies, this section uses the six MOEAs above to optimize instances used S2, which are also used in Section 5.4. The results are shown in Table 4.

**Table 4:** Effects of strategies on the performance indicators.

	HV		GD		IGD		RNI (%)	
	S1	S2	S1	S2	S1	S2	S1	S2
C20-4-1	1.39E+05	1.37E+05	0.196	0.545	0.108	10.025	97.89	32.18
C20-4-2	2.44E+05	2.39E+05	0	6.692	0	34.395	100	5.35
C20-4-3	1.94E+05	1.93E+05	0	1.053	0	11.538	100	19.23
C20-4-4	2.76E+05	2.69E+05	0	2.674	0	38.075	100	8.89
C30-5-1	6.23E+05	6.14E+05	0	0.748	0	13.720	99.77	0.47
C30-5-2	1.55E+05	1.49E+05	0.083	0.840	0.290	28.118	97.74	4.18
C30-5-3	6.13E+05	6.00E+05	0	0.431	0.007	29.140	99.59	1.64
C30-5-4	3.22E+05	3.20E+05	0.024	1.785	0.077	21.914	98.65	6.97
C40-6-1	6.52E+05	6.41E+05	0.090	0.810	0.189	23.035	97.96	3.70
C40-6-2	1.31E+06	1.29E+06	0.106	0.433	0.189	10.816	97.11	2.89
C40-6-3	1.72E+06	1.70E+06	0	0.676	0	32.450	99.81	1.96
C40-6-4	5.34E+05	5.22E+05	0.098	0.555	0.285	21.805	97.90	4.27

**Fig 5:**
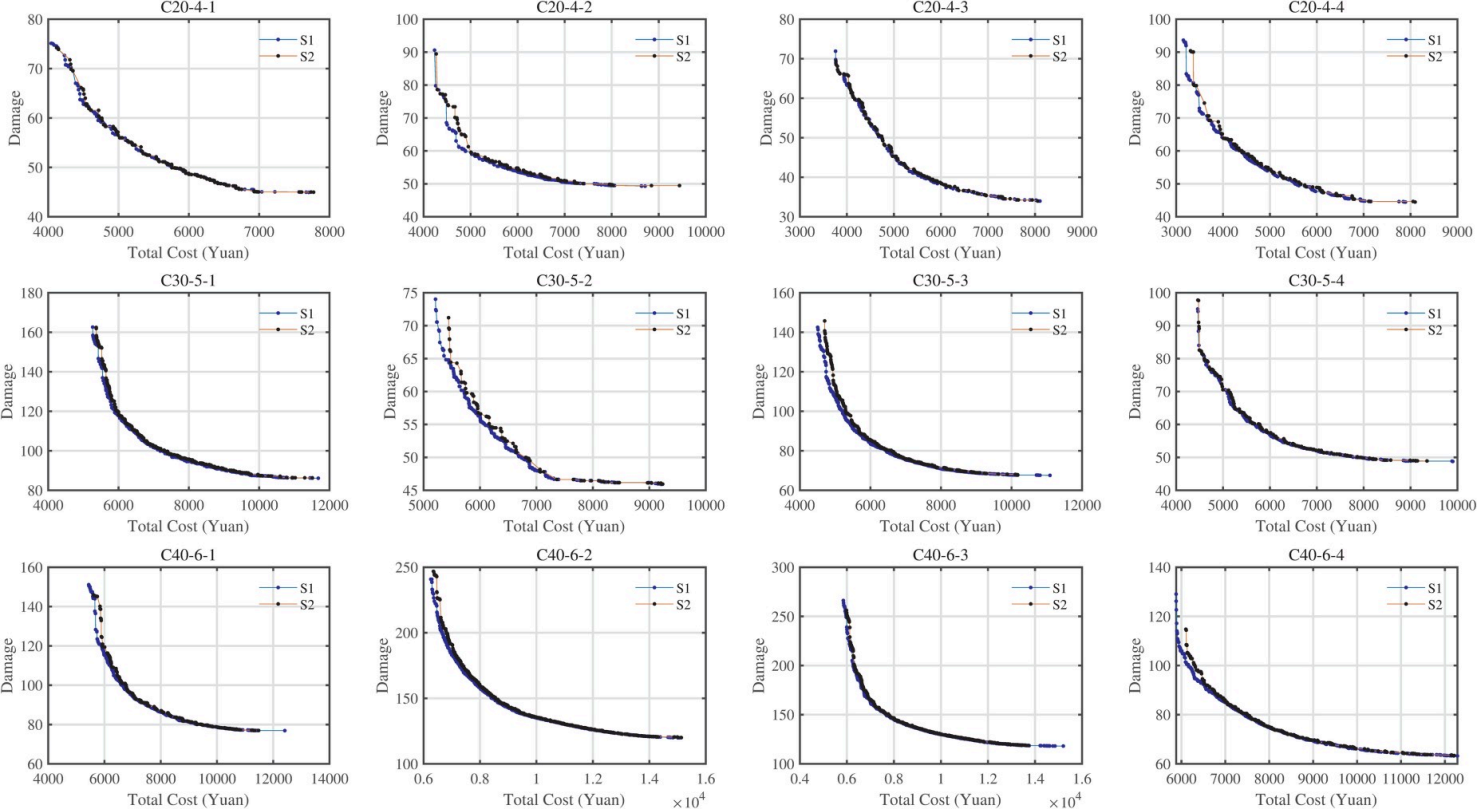
Pareto fronts of instances using S1 and S2.

As shown in Table 4, the performance of S1 is much better than S2, especially C20-4-2, C20-4-3, and C20-4-4. S1 could help increase the HV value by 1.75%, reduce the GD value by 91.62% and the IGD value of 99.46%. Based on the RNI values, S1 and S2 will provide 98% and 0.09% non-dominated solutions for RPF, respectively. The proposed strategy could obtain better Pareto fronts than S2, as shown in Fig 5, and it can be concluded that most of the solutions obtained by S1 dominate the solutions obtained by S2. Therefore, S1 will help provide decision makers with better solutions to choose the right solutions.

Although the positions of Pareto fronts obtained by the two strategies are very close, the RNI indicator can provide the proportion of non-dominant individuals in the construction of RPF. Considering the length of this article, we also provided a partial enlargement of the Pareto frontier in the S1 File and analyzed the impacts of two strategies on CE/fuel consumption, vehicle travel distance, travel time, and total waiting time (VWT). And we could draw the following conclusions for most instances: (1) S1 seems to produce more but not large CE than S2; (2) The travel distance of S1 is less than the travel distance of S2; (3) The travel time of S1 is much lower than the travel time of S2; (4) The VWT of S1 is also much lower than the VWT of S2. Therefore, we conclude that for LRPLCCC, S1 is better than S2.

### 5.6 Efficiency of the proposed model

Since this paper first studied the LRPLCCC, the proposed model utilizing FCCE costs as routing cost should be analyzed and compared with the traditional models using travel distance (TD.) and travel time (TT.) as routing cost. In the models with TD. and TT., we assume that prices per kilometer and minute of both models are 5 Yuan/km and 2.5 Yuan/min, respectively. After the Pareto front of each instance was obtained by the models with TD. and TT., we used the proposed model to recalculate two objective values for comparing the Pareto fronts. Table 5 is the performance indicators for the three models, where all individuals (including the dominated solution) obtained by models using TD and TT were used to recalculate the Pareto fronts under FCCE. In addition, the non-dominated solution obtained by the models with TD and TT are also used to recalculate the Pareto fronts under FCCE, the corresponding data and figures are shown in the S1 File.

**Table 5:** Performance indicators of three models.

	HV^a^			GD			IGD			RNI(%)		
	TD.	TT.	FCCE	TD.	TT.	FCCE	TD.	TT.	FCCE	TD.	TT.	FCCE
C1	1.25	1.33	1.39	3.80	2.13	8.19E-1	44.58	22.60	7.47E-2	3.95	3.98	98.72
C2	2.30	2.30	2.44	4.53	3.37	0	59.65	32.04	0	1.54	2.58	100
C3	1.84	1.91	1.94	2.50	1.17	0	47.94	19.90	0	3.09	18.13	100
C4	2.56	2.63	2.76	3.07	1.34	0	66.24	33.32	3.69E-2	5.58	3.00	99.58
C5	6.84	6.93	7.18	12.36	4.51	1.54E-4	59.07	35.14	2.19	1.66	0.23	99.30
C6	1.58	1.65	1.75	7.21	7.32	0	76.80	36.49	1.94	0.38	0	99.62
C7	5.49	5.75	6.13	2.83	1.91	0	112.72	69.09	1.76E-1	0	0.41	99.59
C8	2.84	3.01	3.22	2.32	5.78	0	134.38	49.67	0	0.34	0	100
C9	5.09	5.88	6.52	8.75	12.24	1.05E-2	380.86	113.98	9.06E-3	0.46	0	99.54
C10	10.28	12.56	13.06	2.17	1.96	0	508.43	28.50	2.33E-2	0.15	0	99.85
C11	14.54	16.54	17.16	2.94	1.63	3.96E-2	257.10	46.70	5.54E-1	0.77	0	99.23
C12	4.32	4.92	5.34	1.85	9.39	3.26E-1	237.15	55.21	6.63E-2	1.05	0	99.16

^a^ The HV values are divided by 10^5^.

**Fig 6:**
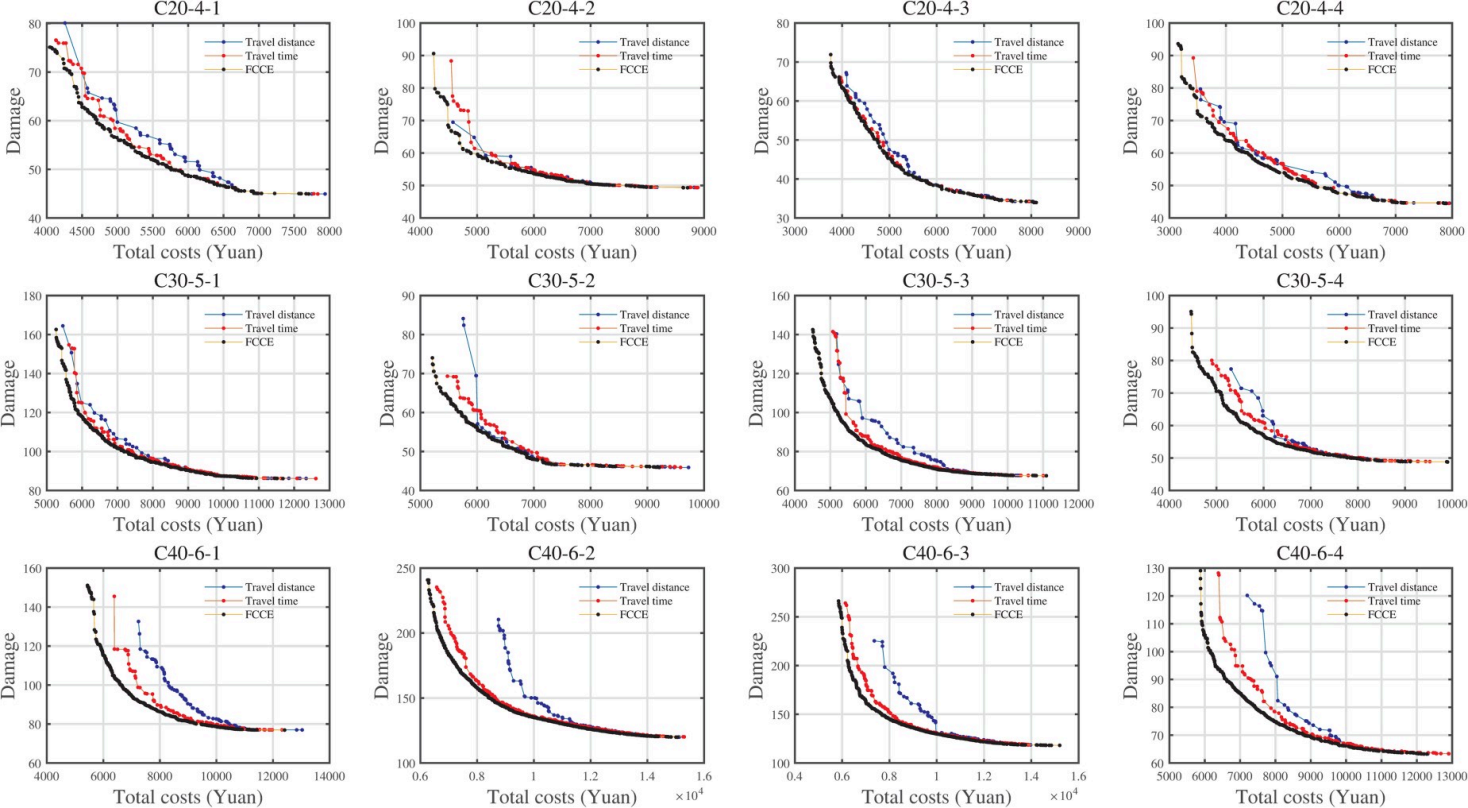
Pareto fronts obtained by three models.

Looking at Table 5, for the HV values, the increment of the Pareto front obtained by the model using the FCCE averaged 14.05% and 5.57% compared to the model with TD. and TT. For the GD and IGD values, the proposed model can reduce the GD values of 96.61% and 96.29%, and the IGD values of 99.43% and 98.86%, respectively, compared with the model with TD and TT. For the RNI values, the models with TD and TT could reduce by 98.41% and 97.63% when compared to the RNI obtained by the proposed model. Hence, the proposed model in this paper is efficient in terms of Pareto solutions. Fig 6 is the Pareto fronts obtained by three models.

From the Pareto fronts shown in Fig 6, we could conclude that the Pareto fronts of the proposed model in this paper could dominate most solutions obtained by the models with TD and TT, especially when the logistics costs are less than 0.5 the maximum costs. Moreover, the Pareto fronts obtained by the model using TT are better than those obtained by the model using TD. Besides, we also analyzed the effects of three models on the CE/fuel consumption, travel distance, and travel time in the S1 File. As the results showed, the following can be obtained for the most instances: (1) The model using FCCE costs as routing cost could produce the minimum CEs among three models, followed by the model with TT; (2) The model using TD could serve clients within the minimum travel distance, which matches its nature, followed by our proposed model; (3) The model using TT could serve the clients within the minimum travel time and VWT, which matches its nature, followed by our proposed model. Hence, the proposed model using the FCCE cost as routing cost could improve economic, environmental, and social effects.

### 5.7 The effect of depot capacity

In the literature, some studies on LRP solved the instances of an unlimited capacitated depots [56]. We now analyze the impact of depot capacity variants on the Pareto frontier. To this end, we have increased the capacity of candidate depots by five times (+ 10, + 20, + 30, + 40 and + 50%) and a variant with unlimited depot capacity. This section applied eight newly generated instances with 50 and 60 clients. Table 6 lists the performance indicators for the eight cases.

**Table 6:** Performance indicators of seven variants of depot capacity.

		Base case	+10%	+20%	+30%	+40%	+50%	Unlimited
C50-7-1	HV^a^	0	56310.849	64541.450	73879.290	76330.541	77216.331	113984.637
	IGD	62.887	22.574	20.279	12.885	11.505	10.822	0
	RNI	0.027	0.094	0.128	0.354	0.485	0.685	1
C50-7-2	HV^a^	0	6564.255	9916.020	77506.232	129476.100	132404.634	180041.485
	IGD	160.826	148.789	146.449	63.098	27.644	21.674	0
	RNI	0.005	0.042	0.044	0.071	0.139	0.526	1
C50-7-3	HV^a^	0	6497.004	30924.099	103879.118	118714.232	120653.185	198597.871
	IGD	126.795	119.606	83.829	41.922	31.353	29.710	0
	RNI	0.052	0.079	0.128	0.173	0.301	0.417	1
C50-7-4	HV^a^	0	51708.518	73816.209	75256.234	76263.216	76753.747	130865.216
	IGD	147.891	78.247	40.803	39.484	38.387	37.432	0
	RNI	0.036	0.044	0.112	0.199	0.275	0.388	1
C60-8-1	HV^a^	0	11450.009	14579.565	18612.925	90170.385	136935.637	243076.760
	IGD	198.291	184.658	176.999	173.445	94.042	46.017	0
	RNI	0.009	0.018	0.090	0.123	0.149	0.257	1
C60-8-2	HV^a^	0	65013.370	75582.601	76807.830	83249.216	117285.169	211078.289
	IGD	148.786	91.777	74.414	71.835	60.187	33.034	0
	RNI	0.024	0.045	0.097	0.184	0.232	0.247	1
C60-8-3	HV^a^	0	17812.484	26224.729	27081.016	149437.982	156408.985	305548.635
	IGD	175.396	150.995	136.212	135.606	46.158	45.775	0
	RNI	0.022	0.082	0.108	0.128	0.167	0.173	1
C60-8-4	HV^a^	0	12200.861	19155.387	70919.389	115710.120	128323.260	246335.144
	IGD	135.721	126.906	119.346	76.477	47.978	38.334	0
	RNI	0.012	0.020	0.030	0.049	0.164	0.200	1

^a^The HV values minus the minimum HV values of seven variants for each instance

**Fig 7:**
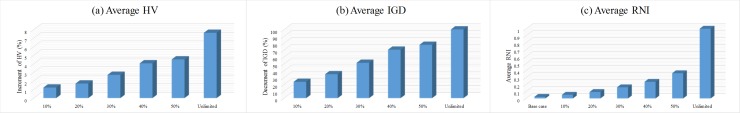
Effects of depot capacity on the performance indicators (only HV, IGD, and RNI).

Looking at Table 6, in terms of HV values, the HV values increase as the depot capacity increases. As shown in Fig 7(A), the increments reach 1.23, 1.70, 2.71, 4.05, 4.51, and 7.61% on average when compared to the HV values of the base instances. As to the average IGD values, the IGD values decrease as the depot capacity increases. As shown in Fig 7(B), the decrements reach 23.74%, 34.76%, 51.39%, 70.54%, 77.60%, and 100% on average when compared to the IGD values of the base instances. From the perspectives of average RNI values, the RNI values increase as the depot capacity increases, as shown in Fig 7(C).

**Fig 8:**
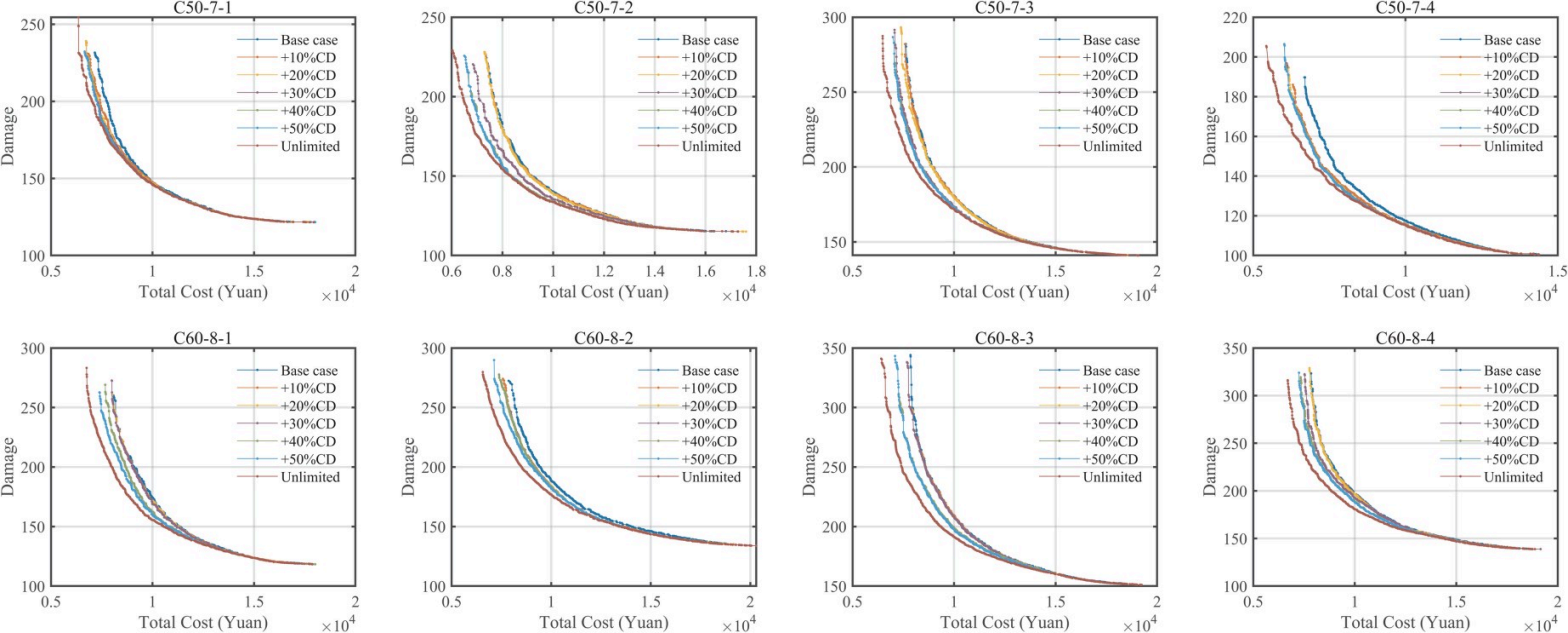
Pareto fronts of seven variants of depot capacity.

Fig 8 is the Pareto fronts of seven variants of the eight instances. We could find that the Pareto fronts of instances with unlimited depot capacity will dominate others. However, the differences between the seven variants are not particularly large. The reason could be hard time windows. The hard time windows for clients and depots limit the LRPLCCC logistics network, so while the depot capacity increases, there are some clients that cannot be served by some depots.

In the S1 File, we also analyzed and compared the effects of the depot capacity on the FCCE, travel distance, travel time, VWT, and total costs of depots to open. As the results of most instances showed that: (1) The exhausted CEs, travel distances, and travel time increase as the depot capacity increases; (2) The total waiting time of vehicles and costs of depots to open decrease as the depot capacity increases. The reason is the higher the depot capacity, the more clients a depot serves, resulting in that fewer depots can be opened and vehicle travel distance, CE, and travel time are increased. While the vehicle waiting time is related to the client time windows, the results showed that the time windows match the cases with unlimited depot capacity.

As stated as Koc et al. (2019) [34], in practice, for some logistics enterprise and their logistics items, the depot capacity is not very important, and our results of the instances with unlimited depot capacity showed that Pareto fronts have better HV, IGD, and RNI values.

### 5.8 The effect of hard time windows

Section 5.7 has analyzed the effects of the depot capacity on the Pareto fronts and several performance indicators of the LRPLCCC, and we could obtain that the hard time windows of clients impact the selection of the sets of the depots to open and the tracings of the routes. Hence, this section is provided to analyze the effects of the time windows of clients on the Pareto fronts and other characteristics of the LRPLCCC. Again, we also generated six variants of the base cases by changing the time window margins like our previous work [28], i.e., *l*_*i*_ = *e*_*i*_+(1+*δ*) ×*st*_*i*_ (*i*∈*N*) where *δ*∈ {10%, 20%, 30%, 40%, 50%}, and a special case *l*_*i*_ = ∞ (i.e., *δ =* ∞). Table 7 presents the results on performance indicators of seven cases, i.e., HV, IGD, and RNI.

**Table 7:** Performance indicators of seven variants of depot capacity.

		Base case	10%	20%	30%	40%	50%	∞
C50-7-1	HV^a^	0	14267.452	17203.765	29451.858	40307.588	57301.647	276095.465
	IGD	182.991	172.624	168.454	157.731	146.948	127.169	0
	RNI	0	0.002	0.015	0.022	0.044	0.090	1
C50-7-2	HV^a^	0	43378.606	48091.871	56427.444	71556.209	77275.918	196577.402
	IGD	108.861	72.340	71.480	61.334	51.751	50.533	0
	RNI	0	0	0	0.019	0.031	0.052	1
C50-7-3	HV^a^	0	64804.477	66511.850	102392.165	114183.324	162281.072	311845.660
	IGD	158.756	111.376	105.997	79.479	66.599	47.390	0
	RNI	0.004	0.011	0.025	0.032	0.141	0.165	1
C50-7-4	HV^a^	0	76998.961	81612.749	89413.921	90921.621	103548.905	293086.959
	IGD	143.785	79.072	75.976	70.481	69.110	60.686	0
	RNI	0.009	0.035	0.035	0.039	0.056	0.058	1
C60-8-1	HV^a^	0	10821.162	59484.107	89643.111	108796.154	137221.322	401376.519
	IGD	215.902	210.434	159.657	137.601	124.188	105.904	0
	RNI	0	0.002	0.008	0.022	0.030	0.049	1
C60-8-2	HV^a^	0	3378.158	5789.026	7042.933	19024.778	32907.905	140136.050
	IGD	43.076	42.008	40.943	36.653	29.824	24.198	0
	RNI	0.181	0.199	0.239	0.445	0.522	0.547	1
C60-8-3	HV^a^	0	62042.357	65775.846	68486.566	74384.570	78554.148	289463.659
	IGD	148.437	92.574	91.904	91.181	86.303	85.775	0
	RNI	0.020	0.027	0.031	0.034	0.053	0.063	1
C60-8-4	HV^a^	0	20713.845	64989.679	67776.946	69954.409	91566.502	328900.033
	IGD	135.142	122.013	87.290	87.033	86.267	73.432	0
	RNI	0.002	0.014	0.036	0.045	0.047	0.118	1

^a^The HV values minus the minimum HV values of seven variants for each instance

**Fig 9:**
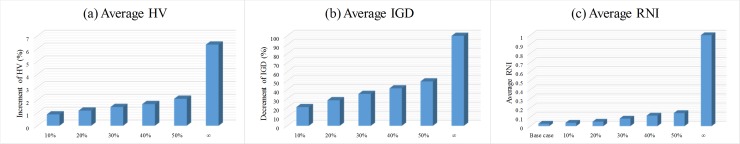
Effects of hard time windows on the performance indicators (only HV, IGD, and RNI).

**Fig 10:**
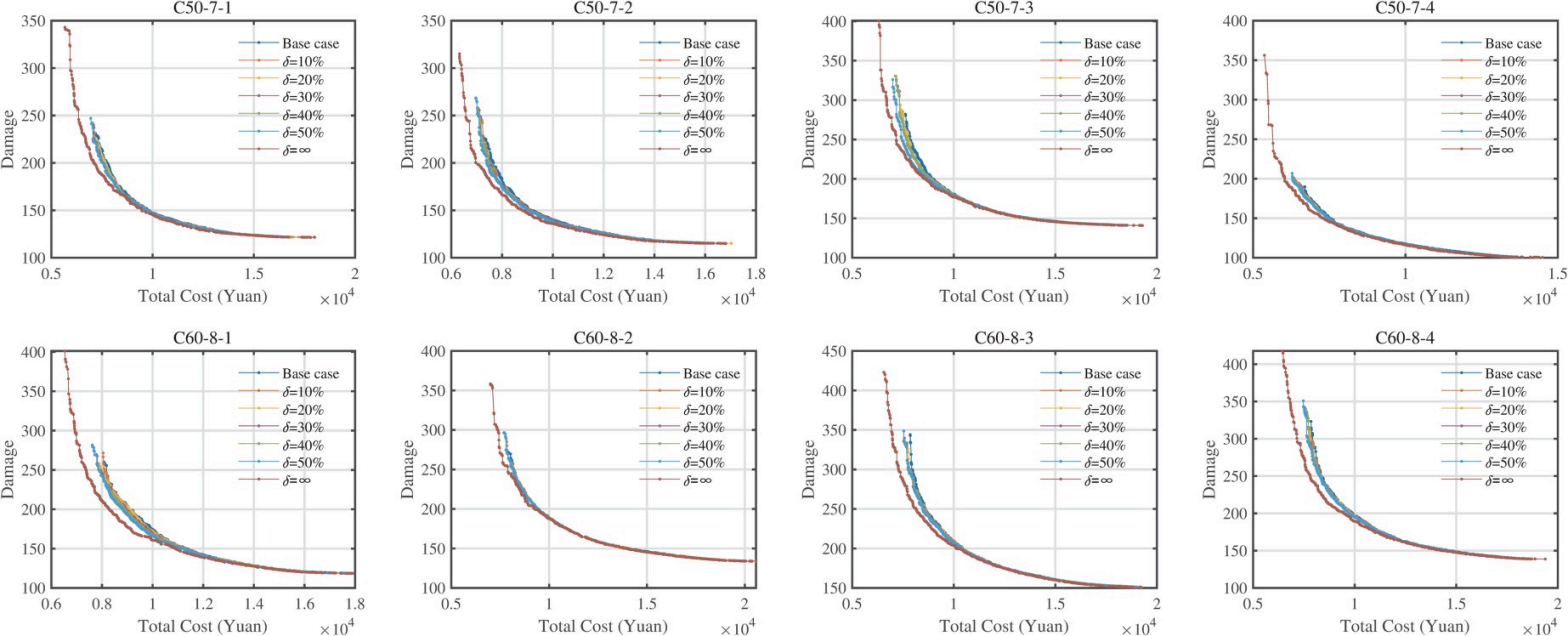
Pareto fronts of seven variants of hard time windows of clients.

Looking at Table 7, we could obtain that: (1) The HV and RNI values of eight cases increase as the *δ* values increase; (2) The IGD values of eight instances reduce as the *δ* values increase. Fig 9 provides comparative analyses on three performance indicators. Six *δ* values could provide an average improvement of 0.88, 1.18, 1.46, 1.69, 2.10, and 6.33% of HV values, and 20.80, 28.40, 35.47, 41.68, 49.33, and 100% of IGD values. In terms of RNI values, six *δ* values could provide 0, 2.69, 3.62, 4.85, 8.24, 11.56, 14.29, and 100% non-dominated solutions for the RPF. Hence, we could conclude that the hard time windows of clients have significant impacts on the performance indicators of the Pareto fronts. Fig 10 provides the Pareto fronts of seven variants of each instances.

As shown in Fig 9, the Pareto fronts of the instances using *δ =* ∞ values dominate most of the solutions of the other cases. As to the others, we could find that there are small (but not large) gaps among the Pareto fronts of the cases using the other six *δ* values. However, from the results in Table 7, larger *δ* values could obtain a better Pareto front, but the range of variation gradually approaches a constant like the results in Section 5.7. Moreover, we could find that the two poles of the Pareto front are extended with the increase of *δ* values, especially when *δ =* ∞. The reason is that the larger closing time window of clients allows much more selection of routings since the closing time window is a strong constraint.

We also analyzed the impacts of the hard time windows on CE/fuel consumption, travel distance, and travel time in the S1 File. Let the second objective be the abscissa, we found that when the damage quality is small (that is, the total cost is large), the above three curves almost coincide, but when the damage quality is large, the curve will change greatly (i.e., the total cost is small), especially CE/fuel consumption, travel distance, and travel time is the smallest in most cases with *δ* = *∞*. Hence, logistics enterprises should analyze the effects of the hard time windows of clients.

Moreover, a novel conclusion could be reached by analyzed the Pareto fronts of this section and Section 5.7: The Pareto front could rotate around a fixed point. This behavior is not shared by most other problems and methods in the literature.

### 5.9 The effect of fleet composition

This section analyzes the benefits of using a heterogeneous fleet of vehicles over a homogenous one. To this end, we have conducted four sets of experiments on the newly generated 50- and 60-client instances, each using a unique vehicle type, i.e., only *h*_1_ (H1), only *h*_2_ (H2), only *h*_3_ (H3), and heterogeneous fleet (HF). The performance indicators of four variants of each instance are presented in Table 8.

**Table 8:** Performance indicators of four variants of fleet composition.

Instance	HV^a^				IGD				GD			
	H1	H2	H3	HF	H1	H2	H3	HF	H1	H2	H3	HF
C50-7-5	265710.26	329596.10	0	452785.26	170.10	30.56	169.57	0	1.78	5.27	70.35	0
C50-7-6	344670.39	517738.20	0	642162.62	109.97	21.96	111.55	0	0.74	6.34	71.88	0
C50-7-7	307479.15	472195.83	0	656261.59	213.51	41.19	160.02	0	1.05	2.39	49.96	0
C50-7-8	377016.12	533627.79	0	667499.85	180.35	23.45	145.20	0	0.92	8.53	85.30	0
C60-8-5	433433.47	515170.52	0	663679.60	144.97	23.50	167.26	0	1.32	4.69	45.63	0
C60-8-6	352120.71	667047.68	0	802593.70	259.37	25.56	164.07	0	2.75	10.05	94.78	0
C60-8-7	421141.24	659051.54	0	788787.80	213.01	34.97	175.58	0	1.28	5.18	42.91	0
C60-8-8	367551.78	522333.36	0	671650.01	138.93	27.95	150	0	1.45	6.83	85.44	0

^a^The HV values minus the minimum HV values of seven variants for each instance

**Fig 11:**
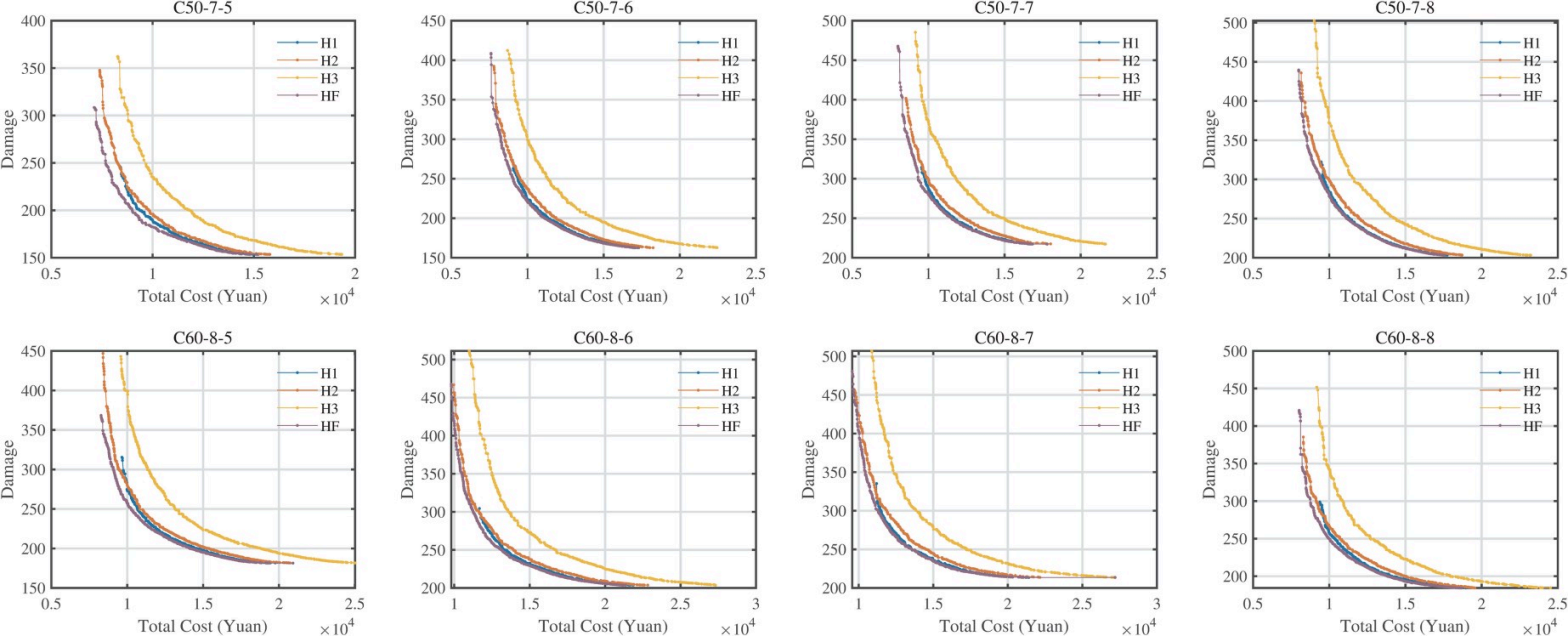
Pareto fronts of four variants of fleet composition.

Looking at Table 8, in terms of the HV values, the instances using HF could obtain the highest values, which provide more 7.66, 3.56, and 18.63% of the HV values compared to the others. From the IGD and GD values, the instances with HF could obtain the best values with all indicators of 0. Fig 11 is the Pareto fronts of eight instances, and the Pareto fronts of the instances using HF could dominate most solutions of other variants. Moreover, the Pareto fronts of the instances using H1 are close to those of instances with HF when the total logistics costs are high, this could obtain from the GD values which are much lower than others. Hence, the HF could provide much better solutions for the decision-makers.

Table 9 is the composition of the vehicle types used in the HF case. We could find that the most commonly used fleet consists of H1 and H2 in the non-dominated solutions of the HF cases, but the combination of H2 and H3 does not provide any non-dominated solutions for the Pareto fronts. Therefore, the instances in this section favor H1 and H2 except for the H2 and H3, other combinations could provide more or less non-dominated solutions. The best fleet composition depends on many factors, such as delivery and pick-up demands and hard time for customers.

**Table 9:** Analysis on the number of solutions with different fleet compositions (a special RNI).

Instance	H1	H2	H3	H1&H2	H1&H3	H2&H3	H1&H2&H3	All Solutions
C50-7-5	0	1.61	0	95.97	0.27	0	2.15	372
C50-7-6	0	0.35	0	98.94	0.71	0	0	565
C50-7-7	4.72	0.47	0	94.10	0.24	0	0.47	424
C50-7-8	16.44	0	0	82.65	0.90	0	0	663
C60-8-5	0	0.18	0	96.29	0	0	3.53	566
C60-8-6	0	1.12	0	97.91	0.96	0	0	623
C60-8-7	1.85	1.85	0.18	93.73	0.55	0	1.85	542
C60-8-8	0	0	0	99.24	0.38	0	0.38	526

In the S1 File, we also analyzed the impacts of the fleet composition on CE/fuel consumption, driving distance, travel time, and total vehicle waiting time. In most instances, the results showed that: (1) The HF in will achieve the lowest CE/fuel consumption, while the use of H3 will bring the highest CE/fuel consumption in the same damage. The reason is that HF's strategy could choose the best types of vehicles for LLRLCCC based on FCCE and vehicle load and total cost. (2) The use of H3 can complete the task at the minimum travel distance, travel time and VWT, which can be explained by the fact that the larger the capacity of the vehicle, the more the vehicle can serve more clients.

Moreover, the limitation of this section is that the fleet composition depends on the nature of the instance, such as vehicle load, hard time window, etc. In other words, if the instance meets the specific requirements, any fleet composition can get the most benefit. However, the generated instances using HF could provide the best Pareto fronts in this paper.

In short, the above sections analyzed efficiency of the proposed strategy and model, and analyzed the effects of the problem parameters such as depot capacity, hard time windows, and fleet composition on the performance indicators of Pareto fronts and LRPLCCC logistics network, such as CE/fuel consumption, travel distance, travel time, and the total waiting time of vehicles. Hence, the logistics enterprises should also analyze the problem parameters on key performance indicators of the logistics network, aiming at improving the sustainable development in economy, environment, and society.

## 6 Concluding remarks

In this work, a novel bi-objective mathematical model for cold chain-based low-carbon location-routing problem was developed. In the proposed model, the first objective consisted of three parts: fixed costs of depots to open, fixed costs of renting vehicles, and the total routing costs, where the latter can be defined with respect to the cost of fuel consumption and carbon emission, and the second objective was used to minimize the total damage on the quality of cargos. Therefore, the bi-objective model can improve economic, environmental and social benefits. This paper also proposes a strategy for mixing cargos, which are the clients’ needs. Besides, several practical constraints were considered in the proposed model: simultaneous pickup and delivery, hard time windows, and a heterogeneous fleet. In the proposed method, we proposed a simple and effective framework inserted in seven well-known multi-objective evolutionary algorithms to solve the proposed model.

In the experiments, we first evaluated the effects of mutational probability in the seven MOEAs, and we determined the top two MOEAs in terms of average HV, IGD, and GD values to optimize the rest experiments. Then, we examined the efficiency of the proposed strategy and model. Extensive analyses are performed to empirically assess the effect of various problem parameters, such as depot capacity, hard time windows, and fleet composition on key performance indicators of Pareto fronts and LRPLCCC network, including fuel consumption, carbon emission, travel distance, travel time, and the total waiting time of vehicles.

This study has some limitations. The model presented in this paper was based on the assumption that the type of cargos for each client remains the same. However, in real-world applications, the client's cargo types could be mixed, i.e., general, refrigerated, and frozen cargos. Hence, the future works may focus on the cold chain logistics considering environmental effects that the types of cargos of each client are mixed. Moreover, the proposed framework randomly selects operators to guide the search rather than adaptively selection based on the performance of each operator. Hence, the future works also may develop a strategy that can monitor the efficiency of operators and adaptively select the promising operators to optimize the problems.

## Supporting information

S1 File (PDF)

S1 Appendix (DOCX)
