# Supplementary material for: A novel bi-objective model of cold chain logistics considering location-routing decision and environmental effects
Source: PLoS One. 2020 Apr 9;15(4):e0230867. doi: 10.1371/journal.pone.0230867 (PMC7145009; doi:10.1371/journal.pone.0230867)
Supplement: S1 File — (PDF) [file pone.0230867.s001.pdf]

Supplementary file of a novel bi-objective model of cold chain logistics considering location-routing decision and environmental effects

Longlong Leng, Jingling Zhang, Chunmiao Zhang, Yanwei Zhao, Wanliang Wang, Gongfa Li

1. **Figures in Section 5.4, that is, the effects of p_m values on the performance indicators**
   See Figs. 1-3
2. **Figures in Section 5.5, that is, the effects of two strategies on CE/fuel consumption, travel distance, travel time, and total waiting time of vehicles (VWT)**
   See Figs. 4-8
3. **Figures in Section 5.6, that is, the effects of three models on CE/fuel consumption, travel distance, travel time, and total waiting time of vehicles (VWT)**
   See Table 1 & Figs. 9-15
4. **Figures in Section 5.7, that is, the effects of seven variants of depot capacity on CE/fuel consumption, travel distance, travel time, and total waiting time of vehicles (VWT)**
   See Figs. 16-20
5. **Figures in Section 5.8, that is, the effects of seven variants of hard time windows of clients on CE/fuel consumption, travel distance, and travel time.**
   See Figs. 21-24
6. **Figures in Section 5.9, that is, the effects of four variants of fleet composition on CE/fuel consumption, travel distance, travel time, and total waiting time of vehicles (VWT)**
   See Figs. 25-28.

1. Figures in Section 5.4, that is, the effects of p_m values on the performance indicators

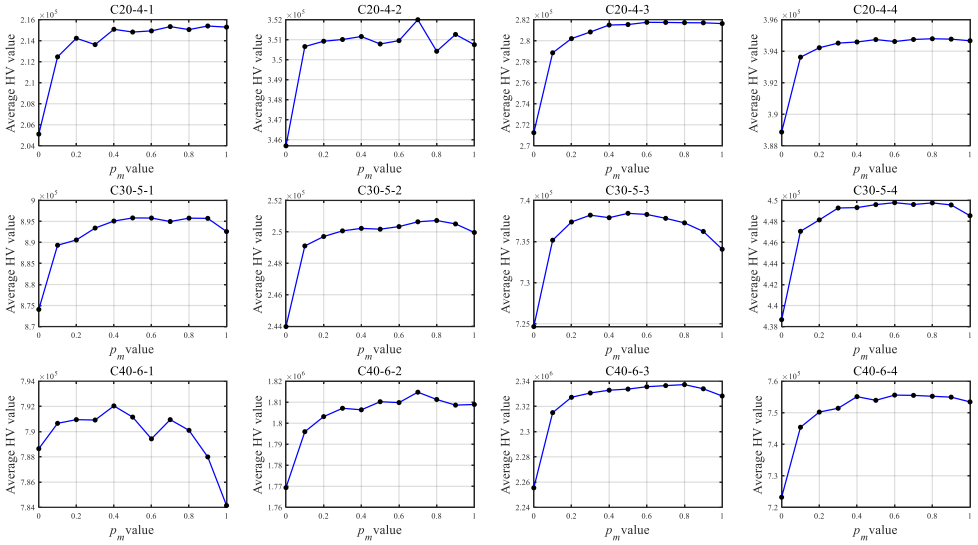

Fig 1. Effects of p_m values on the HV values

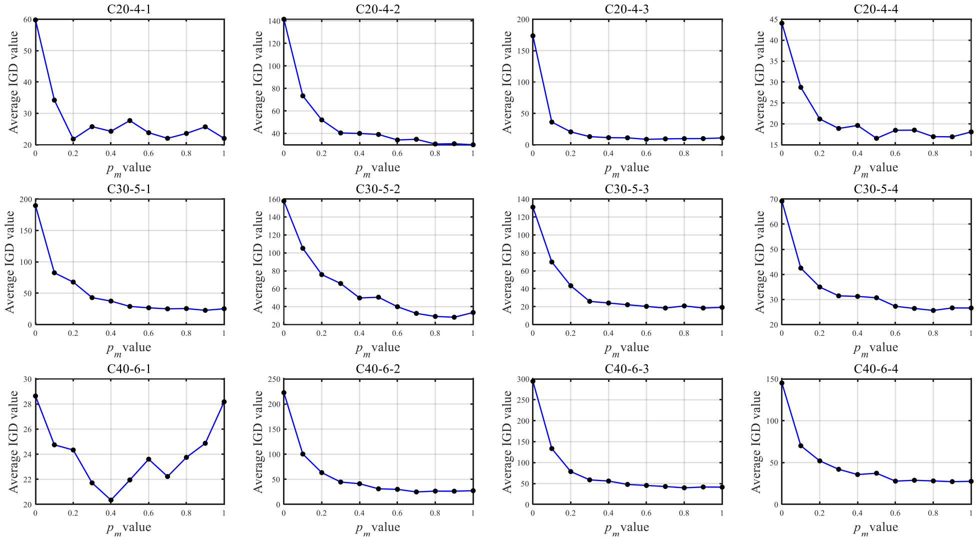

Fig 2. Effects of p_m values on the IGD values

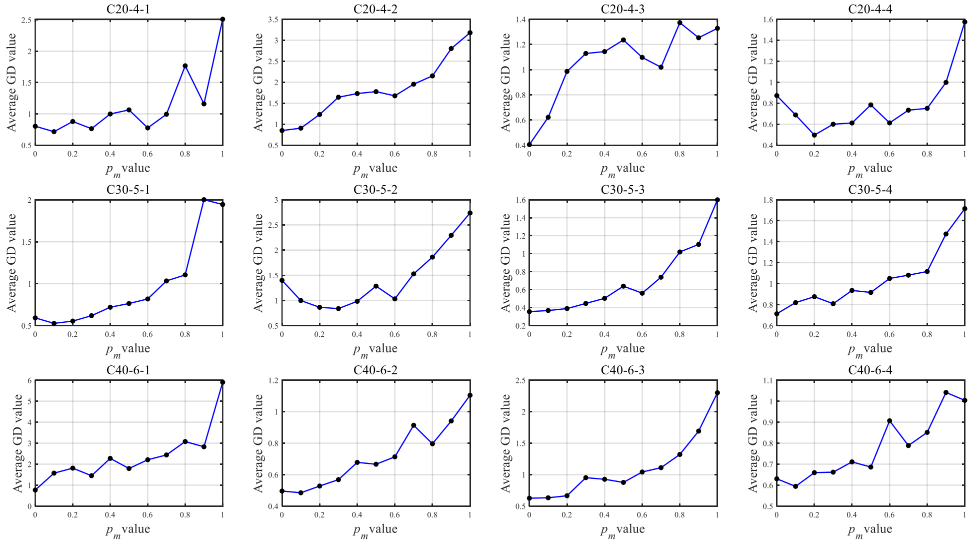

Fig 3. Effects of p_m values on the GD values

2. Figures in Section 5.5, that is, the effects of two strategies on CE/fuel consumption, travel distance, travel time, and total waiting time of vehicles (VWT)

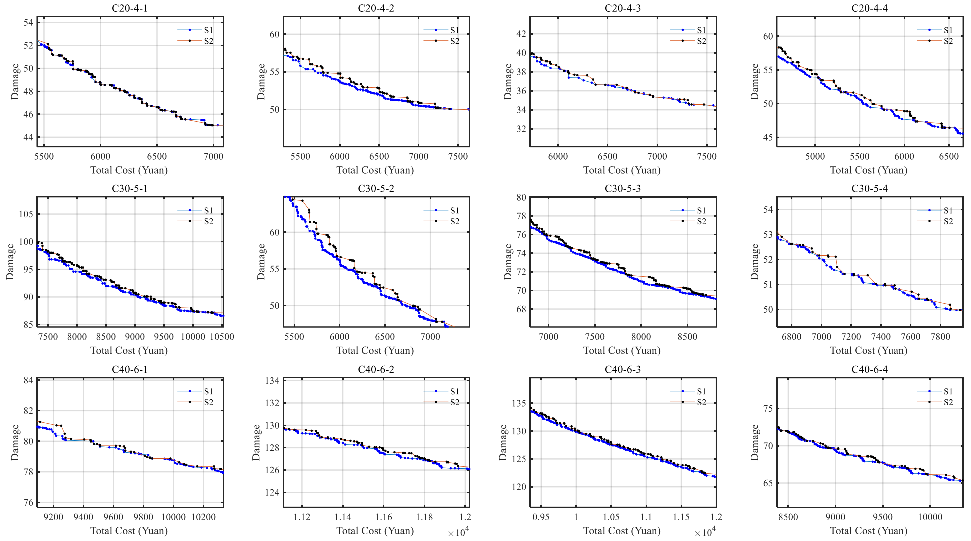

Fig.4 Partial enlargement of Pareto fronts

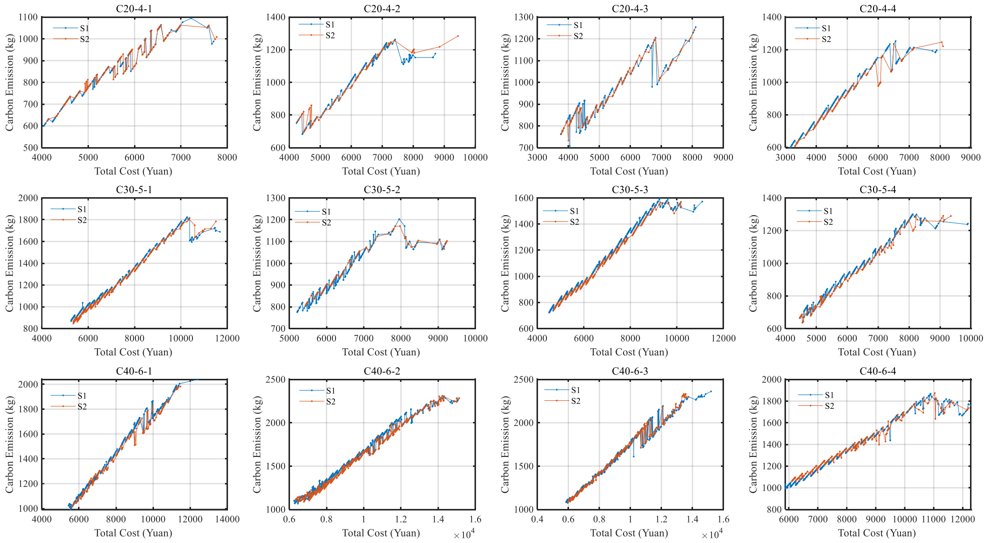

Fig.5 Effects of two strategies on carbon emission (kg)

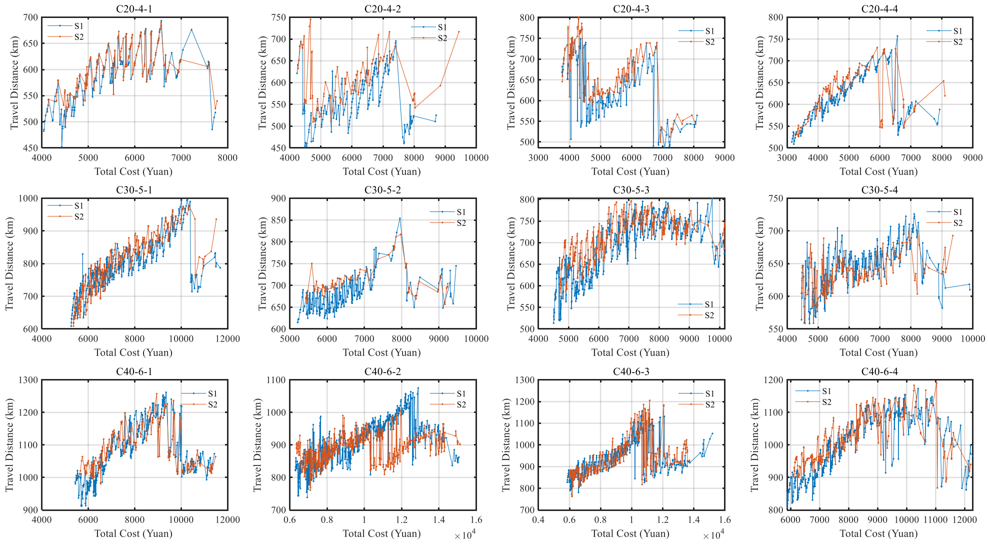

Fig.6 Effects of two strategies on total travel distance (km)

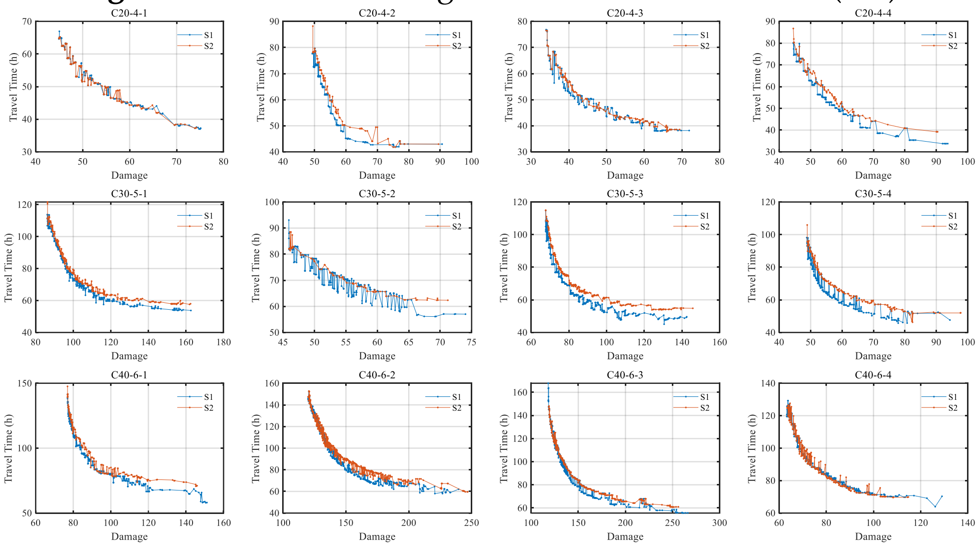

Fig.7 Effects of two strategies on total travel time (h)

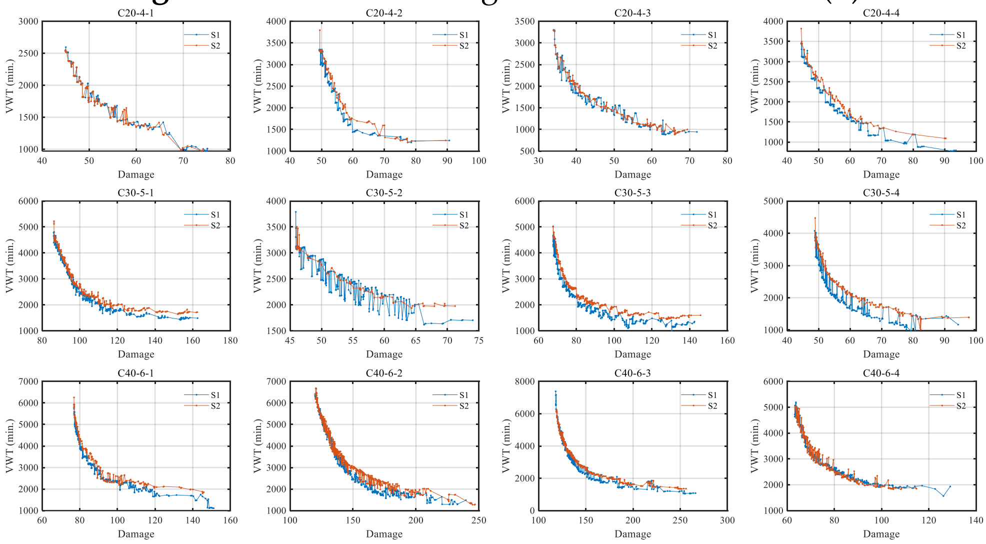

Fig.8 Effects of two strategies on total VWT (min.)

3. Figures in Section 5.6, that is, the effects of three models on CE/fuel consumption, travel distance, travel time, and total waiting time of vehicles (VWT)

Table 1. Performance indicators of three models for the Pareto solutions obtained by three models

	HV			GD			IGD			RNI(%)		
	TD.	TT.	FCCE	TD.	TT.	FCCE	TD.	TT.	FCCE	TD.	TT.	FCCE
C1	1.21E+5	1.27E+5	1.39E+5	5.23	6.69	0.819	92.64	69.10	7.47E-2	3.06	1.30	98.72
C2	2.18E+5	2.20E+5	2.44E+5	4.12	12.59	0	94.25	130.85		0.00	1.03	100
C3	1.78E+5	1.75E+5	1.94E+5	4.54	10.93	0	97.45	196.35		0.51	2.55	100
C4	2.49E+5	2.57E+5	2.76E+5	4.48	3.09	0	67.92	112.80	3.69E-2	4.26	0.84	99.58
C5	6.56E+5	6.49E+5	7.18E+5	23.59	22.58	0	102.58	277.03	2.19	0.23	0.23	99.53
C6	1.55E+5	1.44E+5	1.75E+5	16.66	20.21	0	173.22	151.98	1.94	0.38	0.00	99.62
C7	5.09E+5	5.46E+5	6.13E+5	4.57	14.31	0	244.12	237.34	1.76E-1	0.00	0.41	99.59
C8	2.65E+5	2.78E+5	3.22E+5	4.89	27.40	0	243.05	254.31	0	0.34	0.00	100
C9	4.71E+5	5.43E+5	6.52E+5	15.89	59.84	0	588.60	461.31	0	0.00	0.00	100
C10	1.01E+6	1.23E+6	1.31E+6	2.96	8.46	0	521.83	112.77	2.33E-2	0.15	0.00	99.85
C11	1.36E+6	1.58E+6	1.72E+6	4.36	9.26	0.040	427.76	261.84	5.15E-1	0.58	0.00	99.42
C12	4.16E+5	4.70E+5	5.34E+5	2.18	29.31	0	354.03	192.44	4.62E-1	0.42	0.00	99.58

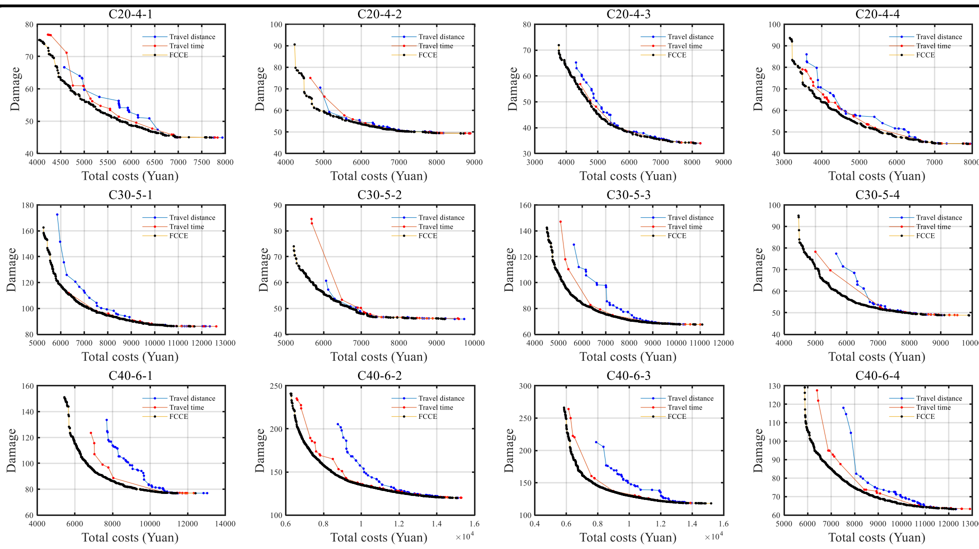

Fig. 9. Pareto fronts under FCCE for the Pareto solutions obtained by three models

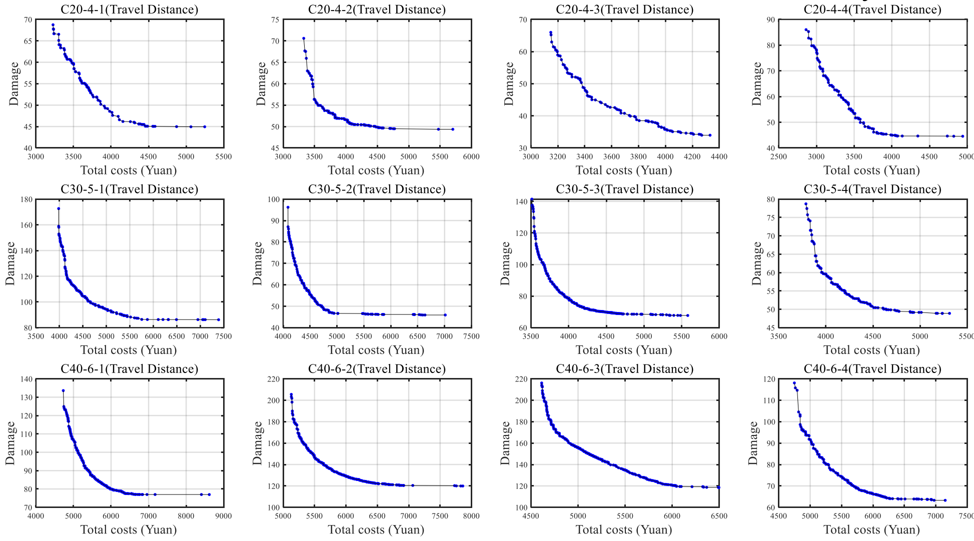

Fig.10. Pareto fronts obtained by the model using travel distance as routing cost (5 Yuan/km)

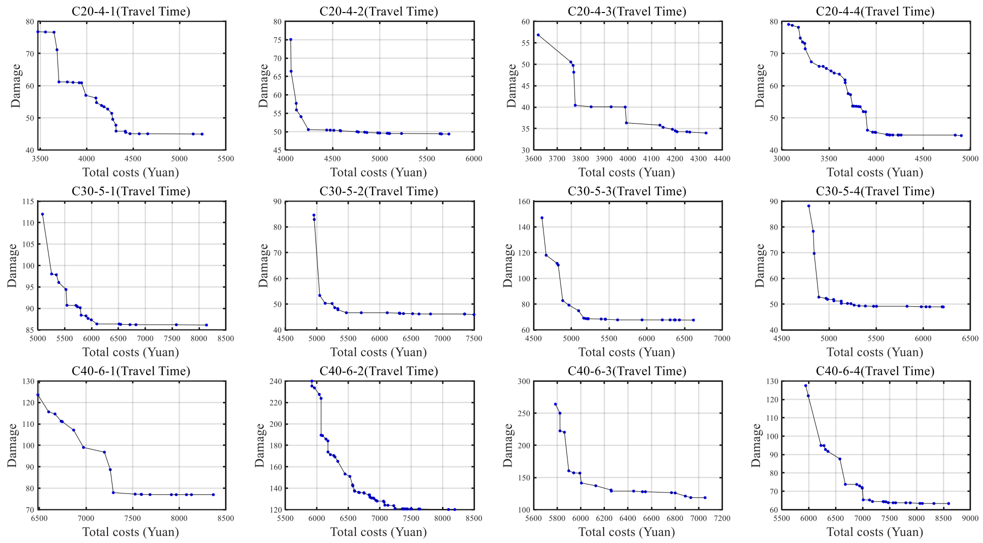

Fig.11. Pareto fronts obtained by the model using travel time as routing cost (2.5 Yuan/min.)

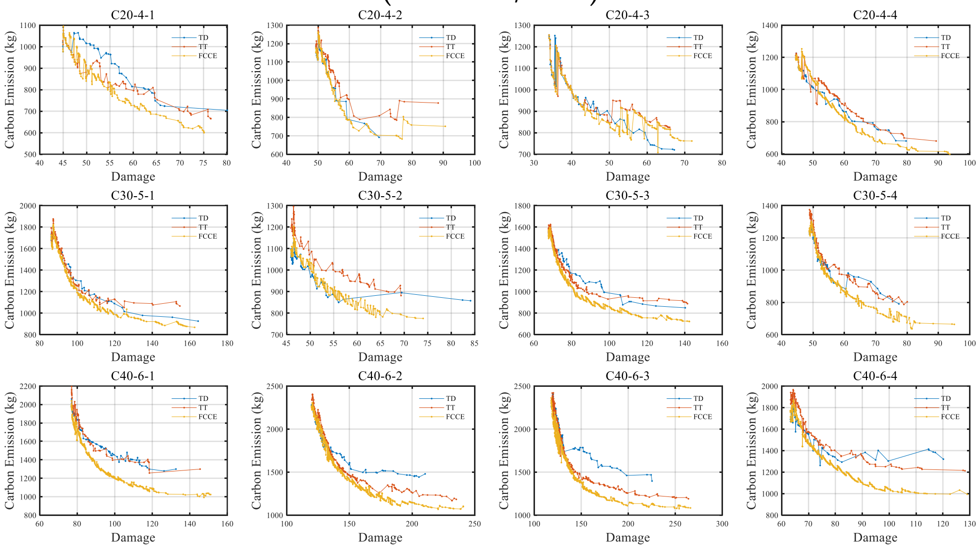

Fig.12. Effects of three models on carbon emissions (kg)

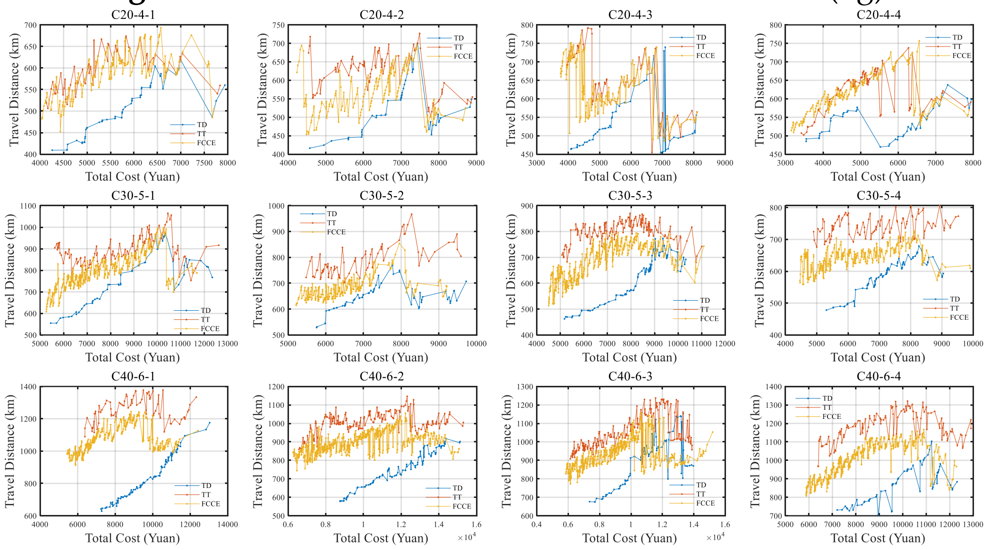

Fig.13. Effects of three models on travel distance (km)

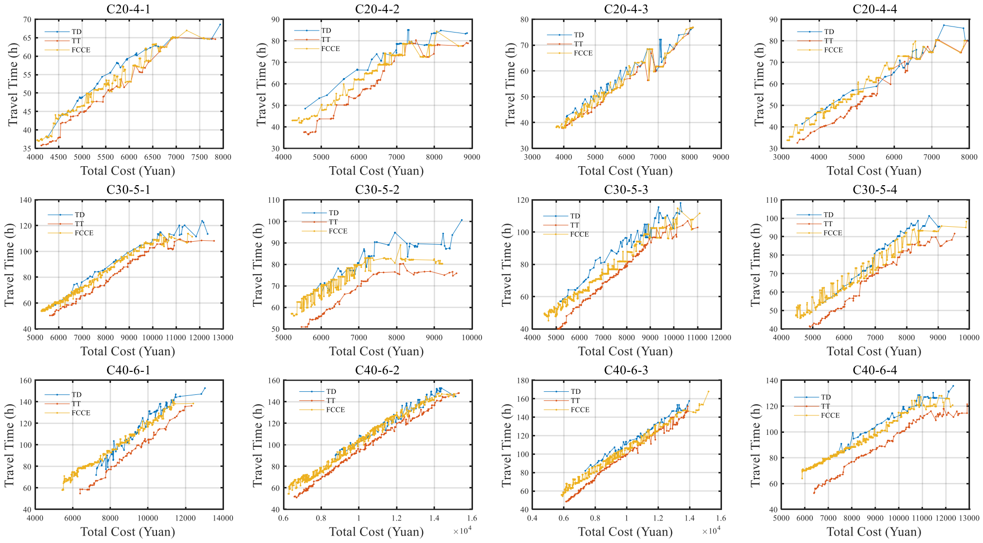

Fig.14. Effects of three models on travel time (h)

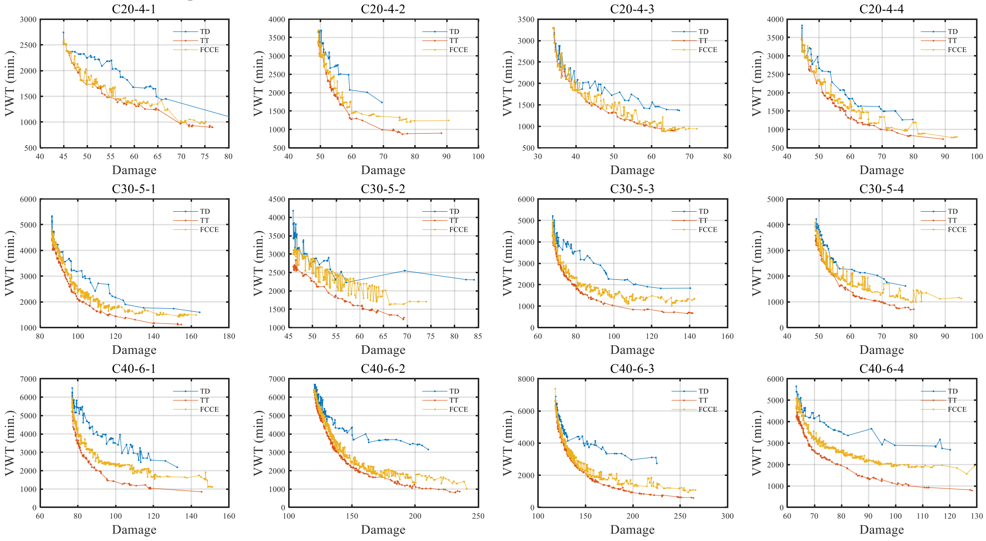

Fig.15. Effects of three models on the total waiting time of vehicles (min.)

4. **Figures in Section 5.7, that is, the effects of seven variants of depot capacity on CE/fuel consumption, travel distance, travel time, and total waiting time of vehicles (VWT)**

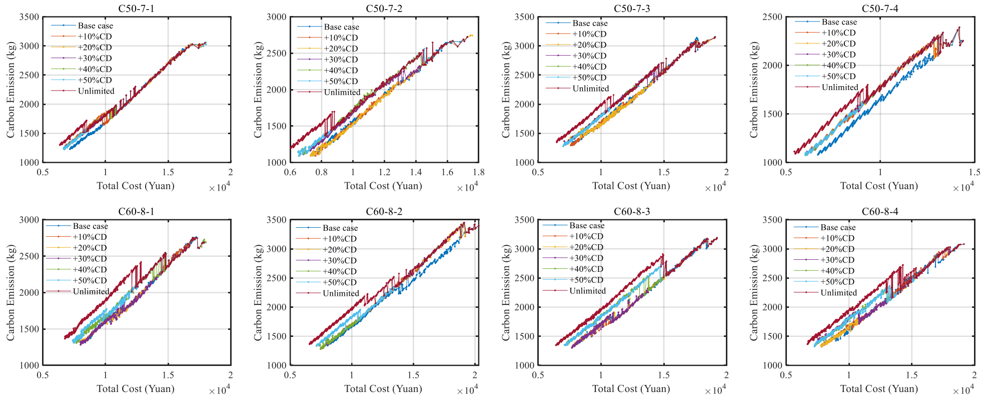

Fig.16. Effects of seven variants of depot capacity on the carbon emissions (kg)

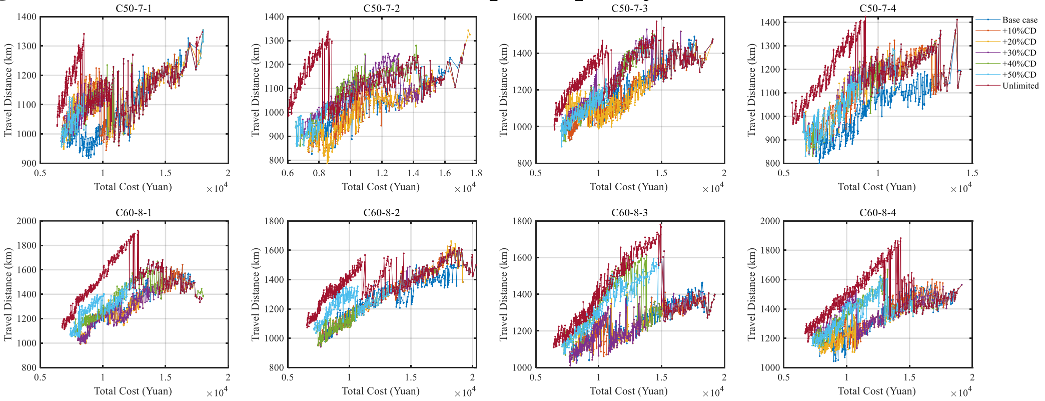

Fig.17. Effects of seven variants of depot capacity on the travel distance (km)

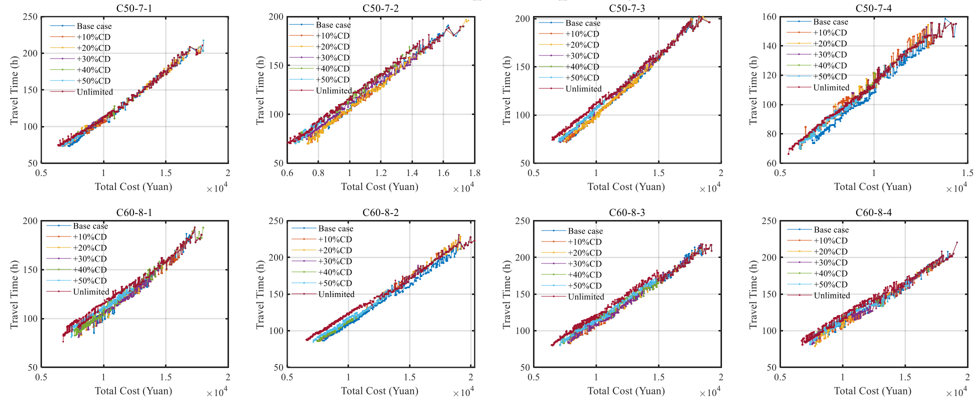

Fig.18. Effects of seven variants of depot capacity on the travel time (h)

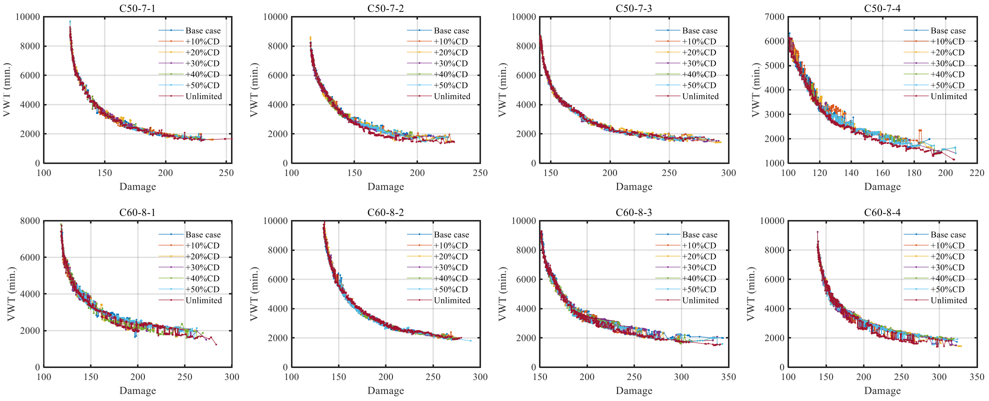

Fig.19. Effects of seven variants of depot capacity on the total waiting time of vehicles (min.)

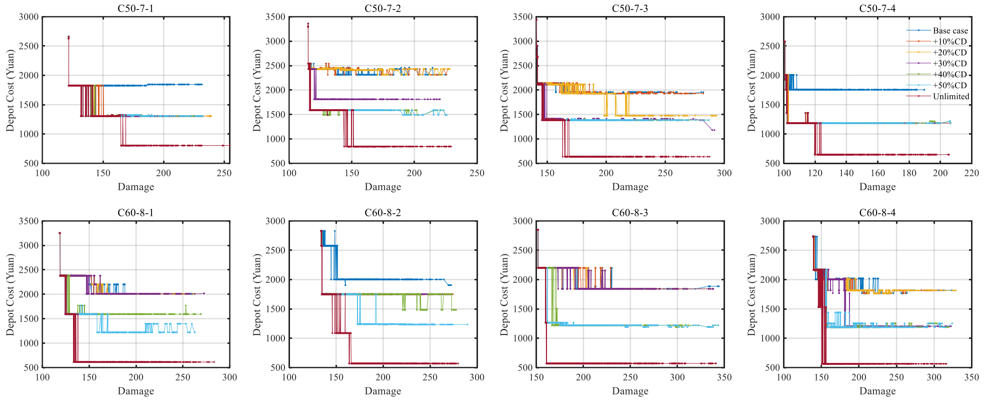

Fig.20. Effects of seven variants of depot capacity on the total costs of depots to open (Yuan)

5. Figures in Section 5.8, that is, the effects of seven variants of hard time windows of clients on CE/fuel consumption, travel distance, and travel time.

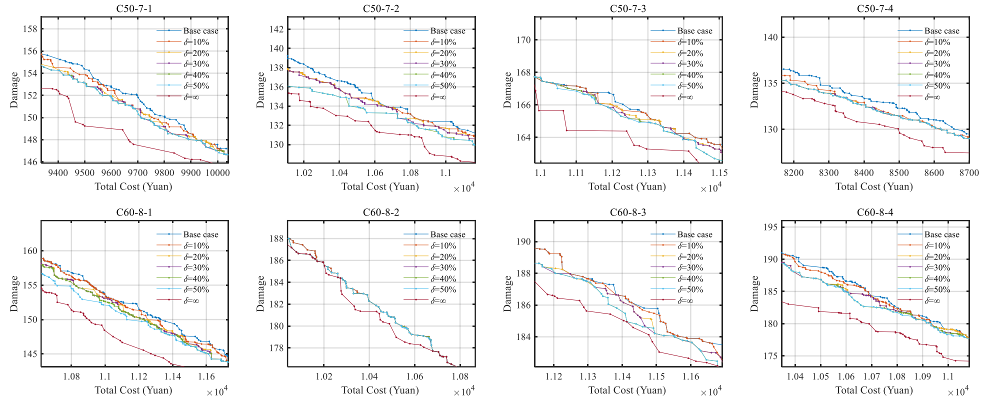

Fig.21. Partial enlargement of Pareto fronts

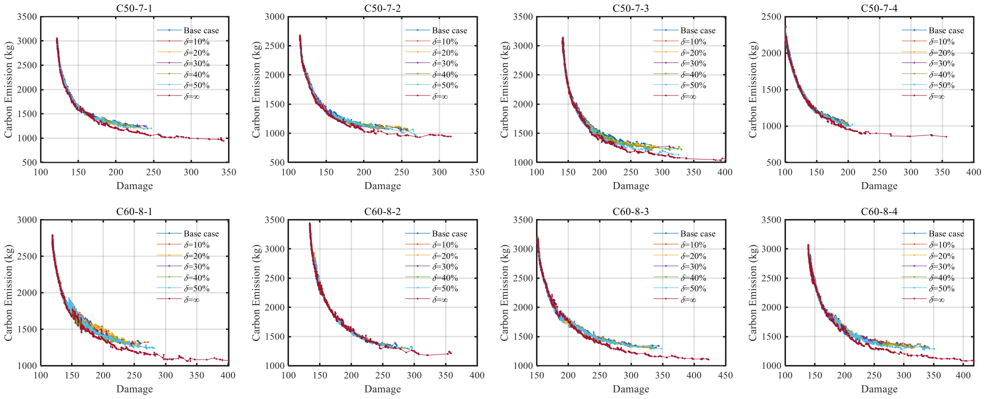

Fig.22. Effects of hard time windows of clients on the carbon emissions (kg)

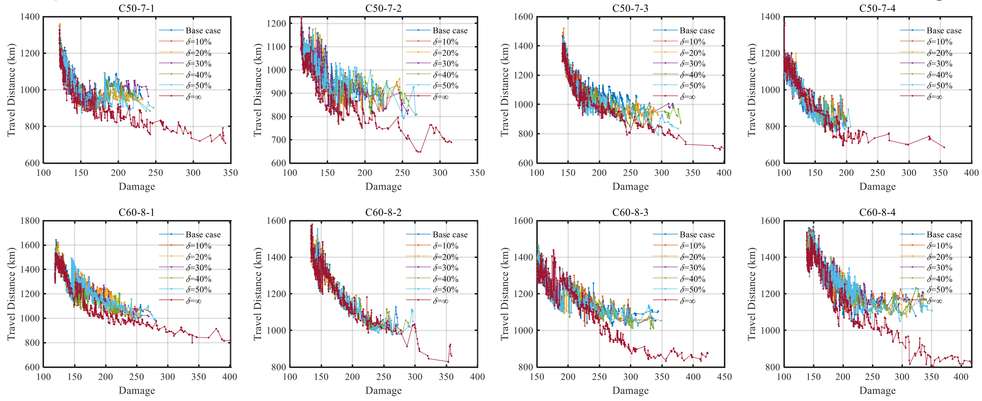

Fig.23. Effects of hard time windows of clients on the travel distance (km)

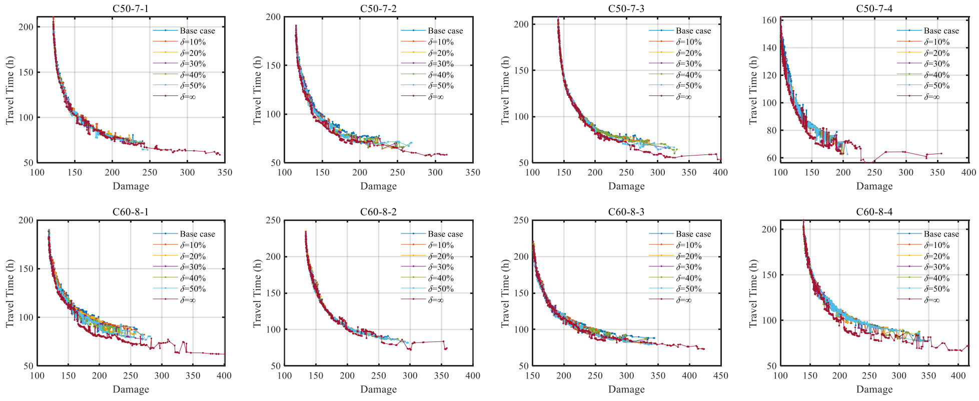

Fig.24. Effects of hard time windows of clients on the travel time (h)

6. **Figures in Section 5.9, that is, the effects of four variants of fleet composition on CE/fuel consumption, travel distance, travel time, and total waiting time of vehicles (VWT)**

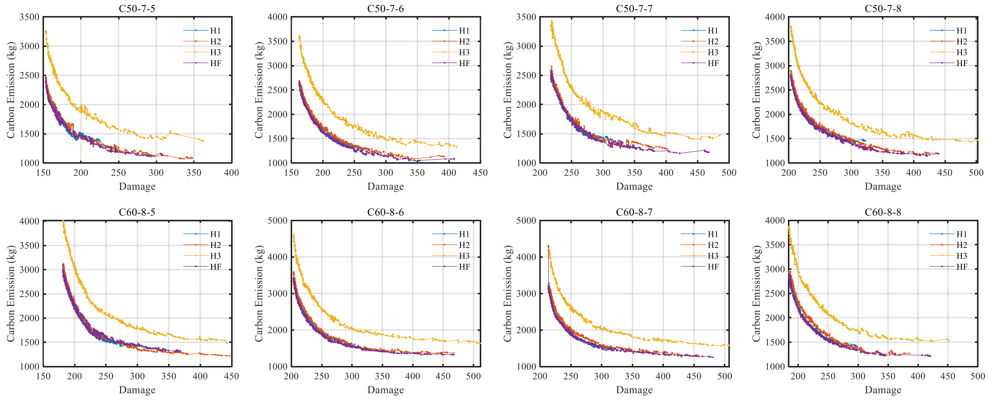

Fig.25. Effects of four variants of fleet composition on the carbon emissions (kg)

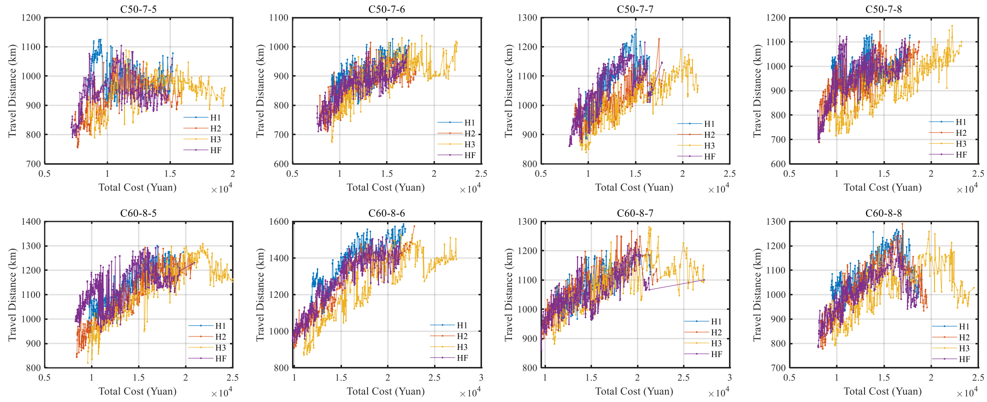

Fig.26. Effects of four variants of fleet composition on the travel distance (km)

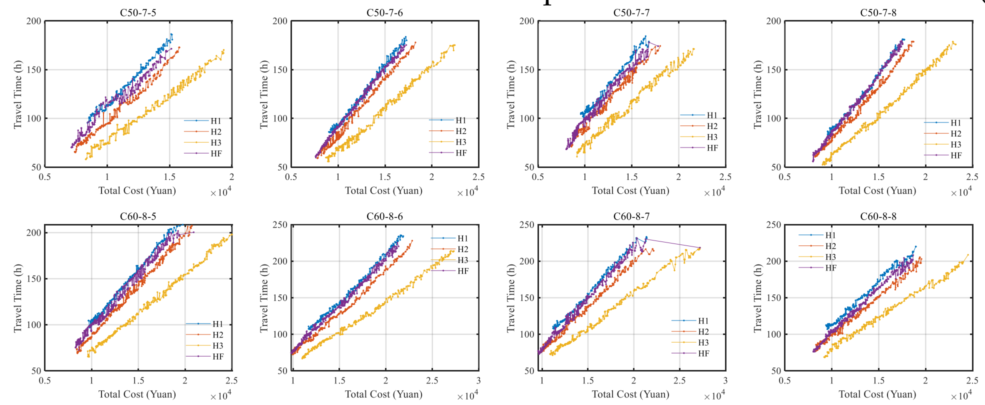

Fig.27. Effects of four variants of fleet composition on the travel time (h)

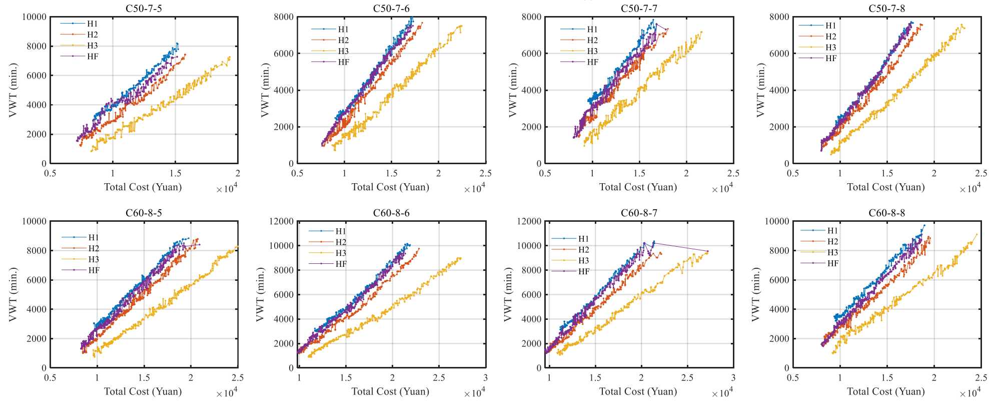

Fig.28. Effects of four variants of fleet composition on the total waiting time of vehicles (min.)
