# Supplementary material for: A novel bi-objective model of cold chain logistics considering location-routing decision and environmental effects
Source: PLoS One. 2020 Apr 9;15(4):e0230867. doi: 10.1371/journal.pone.0230867 (PMC7145009; doi:10.1371/journal.pone.0230867)
Supplement: S1 Appendix — (DOCX) [file pone.0230867.s002.docx]

Supplementary files related to this article can be found at <https://doi.org/xxxxxxxxxxxxx>.
